# A dp53-Dependent Mechanism Involved in Coordinating Tissue Growth in *Drosophila*

**DOI:** 10.1371/journal.pbio.1000566

**Published:** 2010-12-14

**Authors:** Duarte Mesquita, Andrés Dekanty, Marco Milán

**Affiliations:** 1 Institute for Research in Biomedicine (IRB Barcelona), Barcelona, Spain; 2 ICREA, Barcelona, Spain; University of Zurich, Switzerland

## Abstract

Coordination of growth between and within organs contributes to the generation of well-proportioned organs and functionally integrated adults. The mechanisms that help to coordinate the growth between different organs start to be unravelled. However, whether an organ is able to respond in a coordinated manner to local variations in growth caused by developmental or environmental stress and the nature of the underlying molecular mechanisms that contribute to generating well-proportioned adult organs under these circumstances remain largely unknown. By reducing the growth rates of defined territories in the developing wing primordium of *Drosophila*, we present evidence that the tissue responds as a whole and the adjacent cell populations decrease their growth and proliferation rates. This non-autonomous response occurs independently of where growth is affected, and it is functional all throughout development and contributes to generate well-proportioned adult structures. Strikingly, we underscore a central role of *Drosophila* p53 (dp53) and the apoptotic machinery in these processes. While activation of dp53 in the growth-depleted territory mediates the non-autonomous regulation of growth and proliferation rates, effector caspases have a unique role, downstream of dp53, in reducing proliferation rates in adjacent cell populations. These new findings indicate the existence of a stress response mechanism involved in the coordination of tissue growth between adjacent cell populations and that tissue size and cell cycle proliferation can be uncoupled and are independently and non-autonomously regulated by dp53.

## Abstract 2

A study in the *Drosophila* wing suggests a crucial role of p53 in the coordination of growth between adjacent cell populations to maintain organ proportions and shape.

## Author Summary

The coordination of growth within and between organs contributes to the generation of functionally integrated structures and well-proportioned animals and plants. Though these issues have fascinated biologists for centuries, the responsible molecular mechanisms remain largely uncharacterized. In this work, we have used the *Drosophila* wing primordium to show that adjacent cell populations grow and proliferate in a coordinated manner. By reducing growth rates in specific territories within the developing wing, we showed that the tissue responds as a whole and that in adjacent cell populations the growth and cell cycle rates are concomitantly reduced, thus maintaining tissue proportions and normal wing shape. Interestingly, we show that the *Drosophila* tumour suppressor protein dp53 and apoptotic machinery play a key role in coordinating this tissue-wide response. Both growth and proliferation rates are regulated in a coordinated and non-autonomous manner by the activity of dp53, whilst the apoptotic pathway has a specific and non-autonomous role in regulating cell proliferation rates. Our studies describe a novel mechanism for regulating tissue growth in developing organs that may ultimately be relevant for other processes involving coordination of growth, such as tissue renewal, regeneration, and cancer.

## Introduction

In multicellular organisms, coordination of growth between and within organs contributes to the generation of well-proportioned organs and functionally integrated adults. Although the mechanisms that help to coordinate the growth between different organs start to be unraveled [1], the underlying molecular mechanisms that contribute to generating well-proportioned adult organs remain largely unknown.

In *Drosophila*, primordia of the adult head, thorax, and terminalia are established in the embryo as imaginal discs, which grow and proliferate within the feeding larva, fuse during metamorphosis, and give rise to the adult animal [2]. Even though the size, shape, and pattern of each adult structure are genetically determined in an autonomous manner by each imaginal disc, several humoral mechanisms contribute to coordinating the growth between imaginal discs to generate a well-proportioned adult fly. Insulin-like growth factors (IGF) and Target of Rapamycin (TOR) kinase signaling couples the nutritional status of the animal with the growth of each imaginal disc, and steroid and neuropeptide hormones coordinate the termination of growth with developmental timing (reviewed in [3]). Damage or growth retardation of imaginal tissue induces, through the activity of steroid and neuropeptide hormones, a larval developmental delay to ensure that termination of growth is coordinated among growing tissues and all organs attain a characteristic final size [4],[5]. Similarly, the activity of IGFs modulates the growth of each imaginal disc to give rise to well-proportioned adult flies, with variable sizes depending on nutritional status (reviewed in [6]).

Here we used the wing imaginal disc to first analyze whether adjacent cell populations within an organ grow in a coordinated manner to give rise to a well-proportioned structure and afterwards to determine the molecular mediators involved in this coordination. The wing disc is a mono-layered epithelium that grows about a thousand-fold in mass and cell number during larval development. After metamorphosis, it gives rise to the adult wing, a flat structure with a species-specific shape, size, and pattern. By reducing the growth rates of defined territories within the developing wing primordium and analyzing the non-autonomous response throughout development of the adjacent cell populations, we demonstrate that adjacent cell populations respond as a whole by decreasing their growth and proliferation rates. This non-autonomous response occurs independently of where growth is affected, and it is functional throughout development. We underscore a central and non-autonomous role of *Drosophila* p53 (dp53) and the apoptotic machinery in these processes. While the decrease in growth and proliferation rates are regulated in a coordinated and non-autonomous manner by the activity of dp53, effector caspases have a non-autonomous role in reducing proliferation rates. These new findings indicate the existence of a stress response mechanism involved in buffering local variations in growth in order to maintain the relative contribution of each cell population to the final organ and that tissue size and cell cycle proliferation can be uncoupled and are regulated by two different mechanisms downstream of dp53.

## Results

### Adjacent Cell Populations Attain a Final Size in a Coordinated Manner

**Figure 1:**
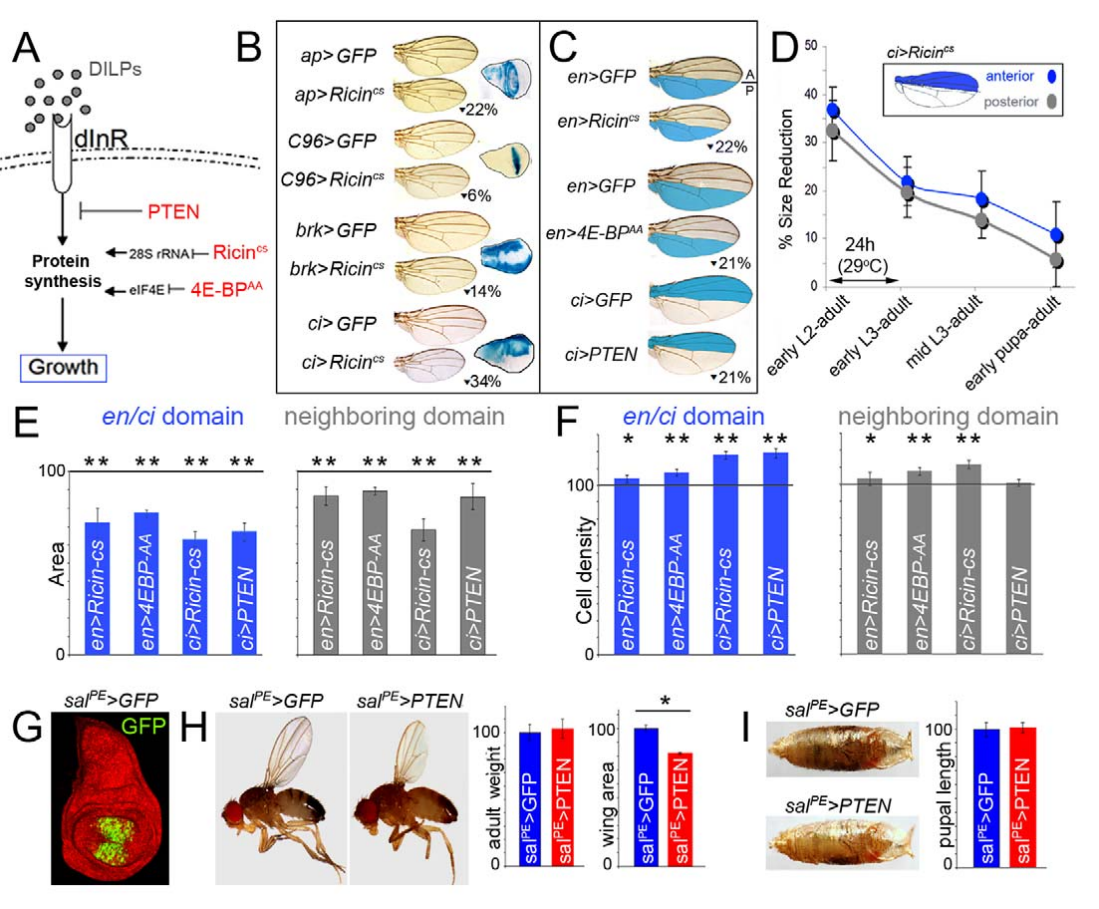
Adjacent cell populations attain a final size in a coordinated manner. (A) Drosophila insulin-like peptides (DILPs) bind to the Insulin Receptor (dInR) and induce tissue growth by increasing the protein biosynthetic capacity of the cells. Three proteins, with well-known growth inhibitory functions in insulin signaling (PTEN) or protein biosynthesis (Ricin^cs^, a cold sensitive version of Ricin-A, and 4E-BP^AA^, an activated form of 4E-BP), were expressed with the Gal4/UAS system in specific territories of the developing wing disc. (B, C) Cuticle preparations of adult wings expressing GFP, Ricin^cs^, 4E-BP^AA^, or PTEN under the control of a range of Gal4 drivers. The reduction in wing size caused by Ricin^cs^, 4E-BP^AA^, or PTEN expression when compared to GFP-expressing wings is shown. Expression domains of Gal4 drivers are shown in wing discs expressing the *UAS-lacZ* transgene and stained for β-Galactosidase activity (blue, A) or depicted in blue (C). Localized transgene expression induced a significant reduction in adult wing size when compared to GFP-expressing wings raised in the same conditions (*p*<10^−4^; see also Table S1). (D) Time-lapse experiments showing the coordinated reduction of tissue size throughout development. Animals expressing GFP or Ricin^cs^ in the *ci-gal4*-expressing domain (A compartment) were transferred to 29°C at different developmental time points until adult eclosion. Ricin^cs^ expression induced a coordinated reduction in tissue size in both A (blue) and P (gray) compartments. The areas of the A and P compartments were significantly reduced when compared to GFP control wings raised at 29°C (*p*<10^−4^; see also Table S4). (E, F) Histograms plotting the size (E) and cell density values (F), normalized as a percent of the control GFP-expressing wing values, of the *en* and *ci* domains expressing different transgenes (blue bars) and of the neighboring domains not expressing the transgene (gray bars). Error bars indicate standard deviation. The horizontal line shows the size or cell density values of the normalized control GFP-expressing wings. Areas were significantly reduced in the transgene-expressing and non-expressing domains (see also Table S2). Cell densities were significantly increased in those bars labeled by one or two asterisks (see also Table S3, * *p*<0.05 and ** *p*<0.01). (G) *sal^PE^*-gal4; *UAS-GFP* wing disc labeled to visualize GFP (green) and DAPI (red). (H,I) Representative *sal^PE^-gal4; UAS-GFP* and *sal^PE^-gal4; UAS-PTEN* adult male flies (H) and pupae (I) with their corresponding histograms plotting the adult weight, wing area, and pupal length, normalized as a percent of the control GFP-expressing wing values. Error bars indicate standard deviation. Adult weight: 100±6 (*sal^PE^ >GFP*) and 103±8 (*sal^PE^ >PTEN*). Wing area: 100±4 (*sal^PE^ >GFP*) and 82±4 (*sal^PE^ >PTEN*). Pupal length: 100±5 (*sal^PE^ >GFP*) and 101±4 (*sal^PE^ >PTEN*). PTEN induced a significant reduction in wing area (*p*<10^−9^), while adult weight (*p* = 0.2) and pupal length (*p* = 0.8) were not significantly changed.

In order to address whether adjacent cell populations grow and acquire a final size in a coordinated manner, we quantified the size and shape of adult wings when tissue growth was reduced in defined territories of the developing wing. Growth, defined as accumulation of cell mass, can be modulated by changing the biosynthetic capacity of cells (reviewed in [7]). In *Drosophila* larvae, starvation of dietary nutrients leads to smaller adult flies. Starvation modulates tissue size by reducing insulin/TOR signaling, which leads to the inhibition of ribosome synthesis, a decrease in nucleolar size, and a reduction in protein synthesis capacity [7],[8]. Thus, a set of genes with well-known growth inhibitory functions in ribosome function, protein biosynthesis, or insulin signaling were expressed with the Gal4/UAS system in specific territories of the developing wing primordium (Figure 1A). A cold-sensitive version of the Ricin toxin A chain (Ricin^cs^), a protein toxin that belongs to the ribosome inactivating proteins that binds and reduces the translational activity of 28S rRNA and thus protein synthesis [9], was used to impair growth, as its activity can be easily modulated in time by changes in temperature [10]. 4E-BP is an important repressor of translation levels and can be inactivated by the protein kinase TOR [11]. 4E-BP binds eIF4E and impairs the recruitment of the 40S ribosomal subunit to the cap structure present at the 5′-end of all eukaryotic cellular mRNAs. To reduce elF4E activity and impair growth, we used a constitutively active form of 4E-BP (4EBP^AA^, [12]) that cannot be phosphorylated by TOR. Finally, the activity of the insulin/TOR pathway was reduced by expression of the tumor suppressor gene PTEN, a conserved negative regulator of the pathway [13],[14]. A collection of Gal4 drivers expressed in distinct domains of the developing wing primordium was used to induce the expression of Ricin^cs^, PTEN, and 4EBP^AA^ (Figures 1B, C and S1). Larvae containing the *Gal4* driver and the *UAS-Ricin^cs^* transgene were initially grown at 18°C (restrictive temperature) until early second instar and then switched to 29°C (permissive temperature) until eclosion of the resulting adult flies. Expression of PTEN and 4EBP^AA^ was achieved by growing the larvae at 25°C. Larvae expressing an *UAS-GFP* transgene and the corresponding *Gal4* driver were subjected to the same temperature schemes and used as controls. Expression of Ricin^cs^, 4EBP^AA^, or PTEN caused a clear reduction in total wing area in all the genotypes analyzed (Figures 1B, C, S1 and Table S1). Interestingly, these wings conserved normal shape, proportions, and vein patterning. The maintenance of shape and wing proportions suggests that the size of the neighboring cell populations not expressing the transgene is reduced in a non-autonomous manner. In order to quantify these non-autonomous effects, we focused our attention on those Gal4 drivers expressed either in the anterior (A, *ci-Gal4*, *ptc-Gal4*, and *dpp-Gal4*) or posterior (P, *en-Gal4*, and *hh-Gal4*) compartments, cell populations that do not mix and give rise to defined structures of the adult wing ([15], e.g. Figure 1C). Both compartments, the one expressing the growth-reducing transgene and the adjacent one, decreased in size (Figures 1E and S1). A size reduction of 11%–14% was observed in the A compartment when Ricin^cs^, 4EBP^AA^, or PTEN were expressed in P cells with the *en-gal4* driver (*p*<10^−3^, Table S2). A similar reduction was detected in the P compartment (14%–32%) when these transgenes were expressed in A cells with the *ci-Gal4* driver (*p*<10^−2^, Table S2). Similar results were obtained with *hh-Gal4*, *ptc-Gal4*, and *dpp-Gal4* drivers (Figure S1). The observed non-autonomous effects in tissue size are most likely a local response to a signal coming from the growth-depleted territory rather than a more general response of the whole larvae induced by the insult to the imaginal cells, since expression of PTEN with a Gal4 driver expressed in the developing wing but not in other tissues (*spalt^PE^-gal4,* [16], Figure 1G) gave rise to smaller wings (82.3%±2.1%; *p*<10^−9^) without a large impact on the overall size of the animal, as visualized by quantification of adult weights (103%±8%; *p* = 0.02) and pupal lengths (101%±4%; *p* = 0.08) (Figure 1G–I and Table S1, see also [4]).

We next took advantage of the temperature sensitivity of the *Ricin^cs^* transgene to conduct time-lapse experiments. Larvae expressing Ricin^cs^ in the A compartment with the *ci-gal4* driver were initially grown at 18°C and switched to 29°C at different developmental stages until adult eclosion. The size of the resulting adult wings was measured. The reduction in size of the nearby P compartment ranged from 5% to 32% depending on when larvae were switched to the restrictive temperature (Figures 1D, S1 and Table S4). Longer exposure to Ricin^cs^ expression gave rise to the strongest phenotypes, thereby suggesting that the non-autonomous effects are operative throughout development (see below). We also noted that the non-autonomous reduction in size of the P compartment was proportional to the reduction in size of the A compartment (Figure 1D).

The non-autonomous reduction in tissue size could be a consequence of reduced cell growth, reduced cell number, or both. To address this issue, we quantified cell densities in the adult wing by counting the number of hairs (each cell differentiates a hair) in a defined area in the two compartments. Cell densities were slightly increased to various degrees in the transgene-expressing compartment (Figures 1F, S1). The increase in cell densities ranged from 1.7% to 19%, depending on the Gal4 driver and the UAS-transgene used (Table S3). In the non-expressing transgene compartment, a significant increase in cell densities was observed mainly in those wing discs with the highest reduction in tissue size (*ci-gal4; UAS-Ricin^cs^*, 111.6%±2.2%; *p*<10^−13^). Thus, changes in cell size contributed in a smaller extent to the non-autonomous reduction in tissue size and mainly in those situations where this reduction was above a certain threshold.

### Targeted Depletion of Growth Induces a Non-Autonomous Reduction in Growth and Proliferation Rates in Adjacent Cell Populations

**Figure 2:**
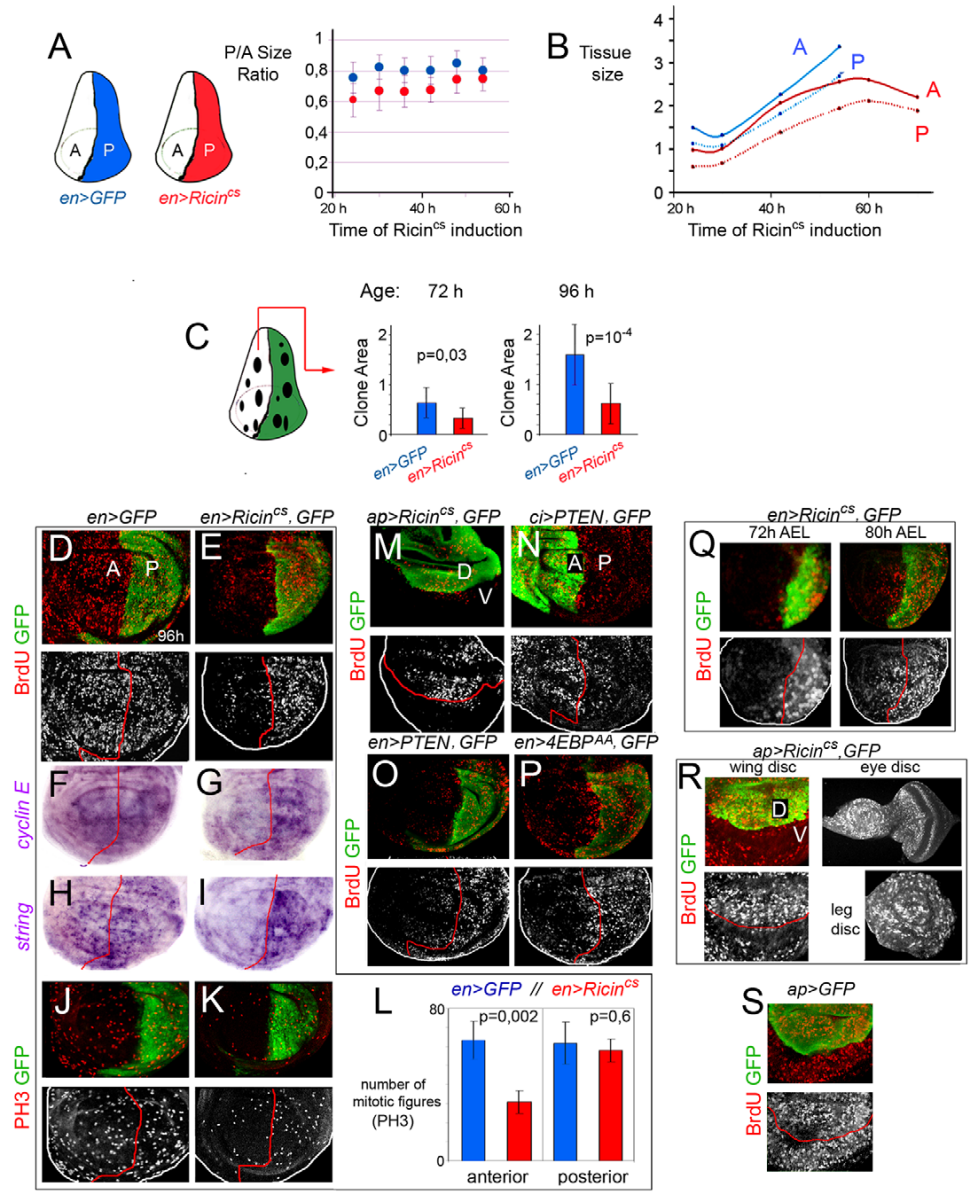
Targeted depletion of growth induces a non-autonomous reduction in growth and proliferation rates in adjacent cell populations. (A, B) Plots representing the size ratio between the P and A compartments (A) and the absolute size (in arbitrary units) of the P and A compartments in *en-gal4;UAS-GFP* (blue dots) and *en-gal4; UAS-Ricin^cs^* (red dots) wing discs raised to 29°C in early second instar and quantified 24–70 h later. P/A ratios (*en>GFP*): 24 h = 0.77±0.1, 30 h = 0.82±0.06, 36 h = 0.80±0.07, 42 h = 0.80±0.1, 48 h = 0.86±0.08, 54 h = 0.80±0.08. P/A ratios (*en> Ricin^cs^*): 24 h = 0.61±0.12, 30 h = 0.67±0.14, 36 h = 0.67±0.11, 42 h = 0.67±0.08, 48 h = 0.75±0.09, 54 h = 0.75±0.09. Size (*en>GFP*): [24 h (A)] = 14±3; [24 h (P)] = 11±2; [30 h (A)] = 13±1; [30 h (P)] = 11±1; [42 h (A)] = 22±3; [42 h (P)] = 18±3; [54 h (A)] = 33±4; [54 h (P)] = 26±4. Size (*en> Ricin^cs^*): [24 h (A)] = 10±3; [24 h (P)] = 6±2; [30 h (A)] = 10±0,3; [30 h (P)] = 6±1; [42 h (A)] = 21±4; [42 h (P)] = 14±3; [54 h (A)] = 25±11; [54 h (P)] = 19±4; [60 h (A)] = 26±9; [60 h (P)] = 21±6; [70 h (A)] = 22±4; [70 h (P)] = 19±3. (C) Histograms plotting the size of clones (in arbitrary units) located in the A compartment of *en-gal4;UAS-GFP* (blue bars) and *en-gal4; UAS-GFP, UAS-Ricin^cs^* (red bars) wing discs. Clones were generated at the beginning of the Ricin^cs^ induction period and quantified 72 or 96 h later in third instar wing discs. Error bars indicate standard deviation. Size of clones at 72 h: *en>GFP* = 0.64±0.3; *en> Ricin^cs^*  = 0.33±0.2. Size of clones at 96 h: *en>GFP* = 1.56±0.6; *en> Ricin^cs^*  = 0.62±0.4. (D–K) *en-gal4; UAS-GFP* (D, F, H, J) and *en-gal4; UAS-GFP, UAS-Ricin^cs^* (E, G, I, K) wing discs labeled to visualize BrdU incorporation (red or white), *cyclin E* or *string* mRNA levels (purple), PH3-positive cells (red or white), and GFP (green). (L) Histograms plotting the number of mitotic figures (PH3-positive cells) observed in the A and P compartments of *en-gal4; UAS-GFP* (blue bars) and *en-gal4; UAS-GFP, UAS-Ricin^cs^* (red bars) wing discs. Error bars indicate standard deviation. Number of PH3-positive cells: *en>GFP*: A = 63±10, P = 62±11; *en> Ricin^cs^*: A = 31±6, P = 58±6. 10 wing discs were scored per genotype. (M–P) Wing discs expressing GFP and *Ricin^cs^* (M), *PTEN* (N, O), or *4E-BP^AA^* (P) with the *ap-gal4*, *ci-gal4*, or *en-gal4* drivers and labeled to visualize BrdU incorporation (red or white) and GFP (green). (Q, R) Wing discs from 72 h AEL and 80 h AEL third instar larvae (Q) and wing, leg, and eye imaginal disc of the same 96 h AEL third instar larvae (R) expressing *GFP* and *Ricin^cs^* with the *en-gal4* (Q) or *ap-gal4* (R) drivers and labeled to visualize BrdU incorporation (red or white) and GFP (green). (S) Wing disc expressing GFP with the *ap-gal4* driver and labeled to visualize BrdU incorporation (red or white) and GFP (green).

The results presented so far indicate that growth depletion in defined territories of the wing induces a non-autonomous reduction in tissue size in nearby territories. In order to gain insight into whether this non-autonomous response is an active mechanism that takes place throughout development, we monitored the size of the A and P compartments throughout development after targeted expression of Ricin^cs^ in P cells. Larvae expressing Ricin^cs^ or GFP in the P compartment (with the *en-Gal4* driver) were grown at 18°C and switched to 30°C from early second instar to late third instar stages. The size ratio between the transgene-expressing (P) and non-expressing (A) compartment was first quantified at a range of time points after induction of Ricin^cs^ expression. The P/A ratio was roughly maintained throughout the induction period in GFP-expressing wing discs (blue dots in Figure 2A). In Ricin^cs^-expressing discs, this ratio was smaller in the first 48 h after transgene expression but reached a similar value in mature wing discs (red dots in Figure 2A, see also [17]). These results suggest that during development the Ricin^cs^-expressing P compartment was relatively smaller than the nearby compartment, but both compartments reached a similar size ratio to that observed in control wing discs at the end of larval development. We next quantified and compared the absolute size of both compartments in GFP- and Ricin^cs^-expressing wing discs. Interestingly, not only was the Ricin^cs^-expressing P compartment already smaller 24 h after transgene expression, but also the adjacent A compartment showed a decrease in size when compared to GFP control wing discs (Figure 2B). As observed in adult wings, the A and P compartments of Ricin^cs^-expressing mature wing discs were smaller than those of control GFP-expressing wing discs (Figure 2B), even though the Ricin^cs^-expressing animals extended their larval period for about 24 h before entering metamorphosis (Figure S2).

We next induced neutral clones of cells at the beginning of the Ricin^cs^ induction period (early second instar) and examined the size of these clones 72 and 96 h later in third instar wing discs. The size of the clones in the A compartment was measured in *en-gal4; UAS-Ricin^cs^* wing discs and was compared to the size of control clones induced in the A compartment of *en-gal4; UAS-GFP* wing discs subjected to the same temperature schemes. In Ricin^cs^-expressing wing discs, the size of the clones visualized 72 or 96 h after clone induction was, respectively, one-half or two-thirds smaller than the size of clones quantified in GFP-expressing discs (Figure 2C). All together, the results presented so far indicate that growth rates are non-autonomously reduced when growth is depleted in defined territories of the developing wing.

In order to gain insight into whether cell proliferation rates are also regulated in a non-autonomous manner, we analyzed cell cycle progression in developing wing primordia exposed to transgene expression. Larvae expressing Ricin^cs^ in the P compartment (with the *en-Gal4* driver) were grown at 30°C from early second (48 h AEL) to mid-late third instar stages (96 h AEL) and the expression of markers for each cell cycle stage was then analyzed. Larvae expressing GFP in the P compartment were subjected to the same temperature schemes and used as controls. S phase progression was first monitored. Imaginal discs were exposed to BrdU for 45 min, and its incorporation was subsequently analyzed. BrdU incorporation was strongly reduced in the adjacent A compartment, while it was not significantly affected in the Ricin^cs^-expressing domain (Figure 2D, E). Similar non-autonomous effects in BrdU incorporation were observed by expression of PTEN or 4E-BP^AA^ in the A or P compartment (with the *en-Gal4* and *ci-Gal4* drivers, respectively; Figure 2N–P). Consistent with the observation that the non-autonomous effects in tissue size were not exclusive to the A and P compartments (Figure 1), a non-autonomous reduction in BrdU incorporation levels was also observed when driving expression of Ricin^cs^ in other domains in the wing primordium (Figures 2M and S2). Similarly, the non-autonomous reduction in BrdU incorporation were also observed at earlier stages of wing development (Figure 2Q), thereby indicating that the non-autonomous effects in proliferation rates were operative throughout development (Figure 1D). The observed non-autonomous effects in BrdU incorporation are most likely a local response to a signal coming from the growth-depleted territory rather than a more general response of the whole larvae, since targeted expression of Ricin^cs^ with the *ap-Gal4* driver, which is expressed in the dorsal compartment of the developing wing, caused a non-autonomous reduction in BrdU incorporation in wing cells while other imaginal tissues not expressing the transgene (e.g. eye) or expressing it late in development (e.g. leg) incorporated BrdU at normal levels (Figure 2R, S).

Next, using in situ hybridization, we monitored the expression levels of *cyclin E* (*cycE*) and *string* (*stg,* the *Drosophila cdc25* homolog), two genes that act in wing disc cells as rate-limiting factors of G1/S and G2/M transitions, respectively [18]–[20]. Consistent with the non-autonomous reduction in BrdU incorporation levels, *cycE* mRNA levels were reduced in the A compartment of wing discs expressing Ricin^cs^ in P cells (with the *en-Gal4* driver, Figure 2F, G). Interestingly, *stg* mRNA levels were also reduced in these cells (Figure 2H, I), suggesting that the G2/M transition was also compromised or delayed in a non-autonomous manner. Consistent with this view, mitotic activity, monitored with an antibody against a phosphorylated form of histone H3 at serine 10 (PH3) that labels mitotic figures, was reduced in these cells (Figure 2J, K). The number of PH3-positive cells observed in the A compartment of *Ricin^cs^*-expressing wing discs decreased by 50% compared to GFP-expressing discs (*p* = 0.02, Figure 2L), whereas a similar number of PH3-positive cells was observed in the P compartment of both genotypes (*p* = 0.6, Figure 2L). Although we noted a slight increase in *stg* and *cycE* mRNA levels in cells expressing Ricin^cs^ (Figure 2G, I), no detectable changes in mitotic activity or BrdU incorporation were observed (Figure 2E, J, L).

The non-autonomous reduction in mitotic activity, BrdU incorporation, and *cycE* and *stg* mRNA levels observed suggest that a general reduction in proliferation rates, without any obvious arrest in any particular cell cycle stage, is non-autonomously induced when growth is impaired in a defined territory of the developing wing. In order to confirm this hypothesis, we used a fluorescence-associated cell sorter (FACS) to collect data about the cellular DNA content of dissociated cells from 96 h AEL wing discs and analyzed the cell cycle profile of these cells. A forward scatter (FSC) analysis was also carried out to compare cell sizes. Cell cycle profiles and cell sizes of A cells dissociated from wing discs expressing Ricin^cs^ and GFP or GFP alone (with the *en-Gal4* driver) in the P compartment were very similar (Figure S2). Comparable results were obtained with the *ci-Gal4* driver (Figure S2). All together these data indicate that upon growth depletion in defined territories of the wing primordium, the adjacent cell populations reduced their growth and proliferation rates, giving rise to smaller structures with a smaller number of cells. The slight non-autonomous reduction in cell size contributes to a small extent to the non-autonomous reduction in adult tissue size (Figure 1E, F) and is most probably occurring during post-larval stages, since no major change in cell size was observed in imaginal tissues (Figure S2).

We noticed that the levels of BrdU incorporation, *stg* and *cycE* expression, and mitotic activity were largely unaffected in the transgene-expressing compartment when compared to GFP control wing discs (Figure 2). This might reflect a process of compensatory proliferation due to the large number of cells being lost by cell death (see below) or by other means.

**Figure 3:**
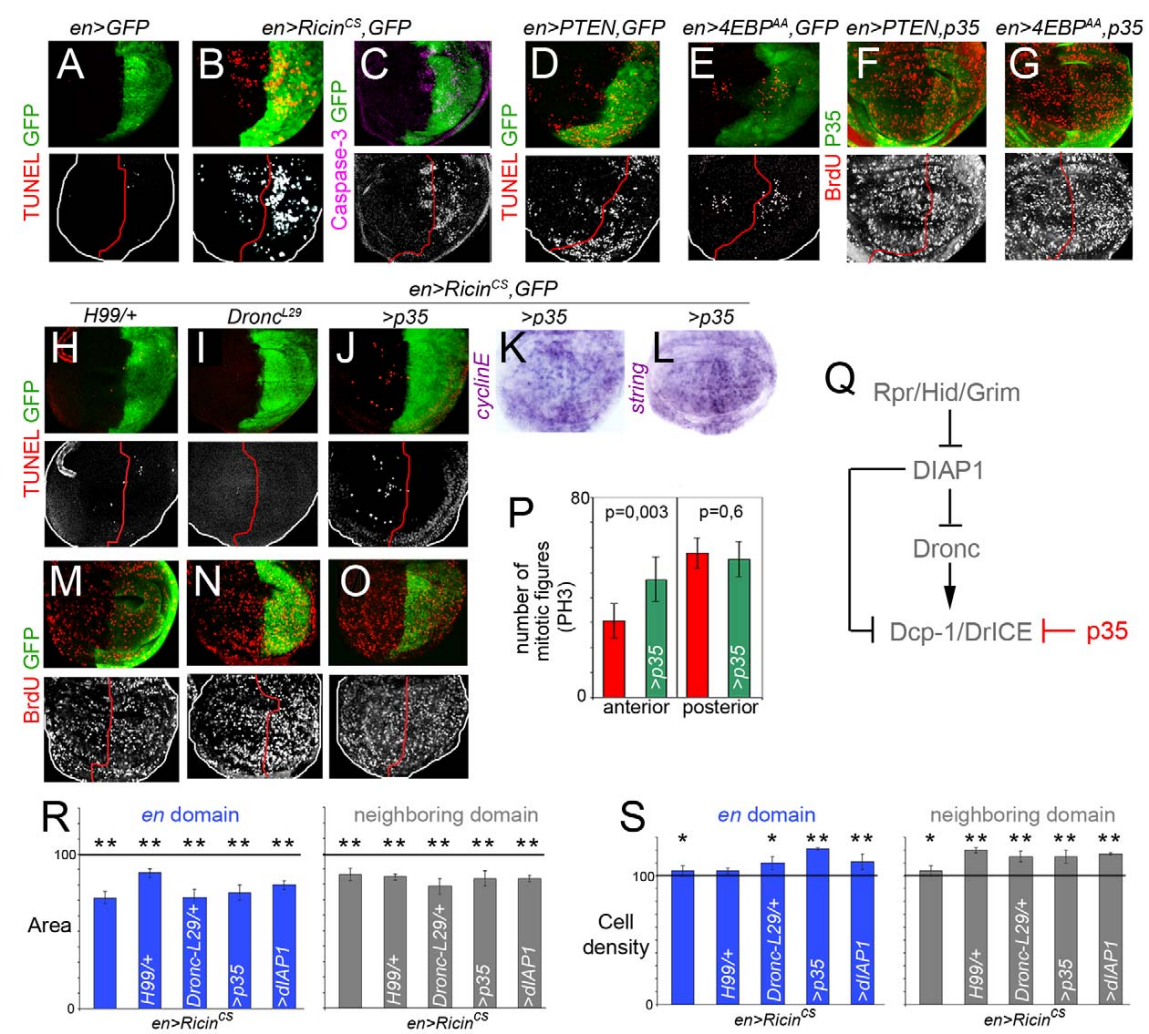
A non-autonomous role of effector caspases in regulating proliferation rates. (A–G) Wing discs expressing GFP alone (A), GFP and *Ricin^cs^* (B, C), GFP and *PTEN* (D, F), or GFP and *4E-BP^AA^* (E, G) with the *en-gal4* driver and labeled to visualize GFP, TUNEL-positive cells, levels of activated Caspase-3 or BrdU incorporation. (H–O) Wing discs expressing GFP and *Ricin^cs^* in different genetic backgrounds (*Df(H99)/+* in H, M; *Dronc^L29^/+* in I, N or expressing p35 in J–L, O) and labeled to visualize GFP, TUNEL-positive cells, BrdU incorporation, and *string* or *cyclin E* mRNA expression. (P) Histograms plotting the number of mitotic figures (PH3-positive cells) observed in the A and P compartments of *en-gal4; UAS-GFP*, *UAS-Ricin^cs^* (red bars), and *en-gal4; UAS-Ricin^cs^ UAS-p35* (green bars) wing discs. Error bars indicate standard deviation. Number of PH3-positive cells: *en> Ricin^cs^*: A = 31±6, P = 58±6; *en> Ricin^cs^*,*p35*: A = 47±9, P = 55±7. Ten wing discs were scored per genotype. (Q) Initiator caspase Dronc and effector caspases DrICE and Dcp-1 are negatively regulated by DIAP1, which in turn is inhibited by the activity of the pro-apoptotic genes *hid*, *reaper*, and *grim*. The baculovirus protein p35 blocks the activity of effector caspases. (R, S) Histograms plotting the size (R) and cell density values (S), normalized as a percent of the control GFP-expressing adult wing values, of the *en* domains expressing *Ricin^cs^* (blue bars) and of the neighboring domains not expressing the transgene (grey bars) in different genetic backgrounds. Error bars indicate standard deviation. Areas were significantly reduced in the transgene-expressing and non-expressing domains (*p*<10^−4^; see also Table S2). Cell densities were significantly increased in those bars labeled by asterisks (see also Table S3, * *p*<0.05 and ** *p*<0.01).

### A Non-Autonomous Role of Effector Caspases in Regulating Proliferation Rates

In mammalian cells, inhibition of the insulin/TOR pathway or inhibition of protein biosynthesis increases the activity of the tumor suppressor gene p53 (reviewed in [21]) and induces the activation of the apoptotic machinery that breaks down cells in a highly controlled fashion by the action of caspases, a specialized class of cysteine proteases. Non-apoptotic functions of activated caspases have been previously described, and *Drosophila* and vertebrate caspases have been reported to regulate cell proliferation in various ways (reviewed in [22], see also [23]). Thus, we first monitored the contribution of the apoptotic machinery to the non-autonomous regulation of tissue growth and cell proliferation rates observed in developing wing discs and resulting adult wings.

We performed a TUNEL assay to label DNA strand breaks induced by apoptotic cell death. A clear increase in TUNEL-positive cells was observed in those territories expressing *Ricin^cs^* (Figure 3A, B), PTEN, or 4E-BP^AA^ (Figure 3D, E) as well as in adjacent cells. We next used an antibody against the activated form of human Caspase 3, a marker of Caspase-9-like Dronc activity in *Drosophila* tissues [24]. We observed increased levels of Dronc activity in those territories expressing the transgene as well as in adjacent cells (Figure 3C and unpublished data). The increase in TUNEL-positive cells and Dronc activity raises the question of whether apoptosis participates in the non-autonomous response observed in developing wing primordia and adult wings. Caspase activities are regulated by inhibitor-of-apoptosis proteins (IAPs), and in *Drosophila*, DIAP1 binds and inhibits Dronc and the effector caspases DrICE and Dcp-1 (Figure 3Q, reviewed in [25]). The *Drosophila* pro-apoptotic genes *hid*, *grim*, and *reaper* bind and repress DIAP1, thus alleviating repression of initiator and effector caspases. In order to analyze the contribution of caspases to the non-autonomous effects in proliferation rates, we tested the requirement of various elements of the genetic cascade that drives apoptosis in *Drosophila* (Figure 3Q). When apoptosis was reduced in the whole tissue by halving the dose of *hid*, *grim*, and *reaper* (in *Df(H99)/+* wing discs) or by depleting *dronc* expression (in *dronc^l29^* wing discs), a clear reduction in TUNEL-positive cells was observed upon Ricin^cs^ expression (compare Figure 3B with 3H, I). Interestingly, the non-autonomous reduction in BrdU incorporation levels caused by Ricin^cs^ expression was largely rescued in these discs (Figure 3M, N and compare with Figure 2E). We then expressed *DIAP1* and *p35* in the same domain as Ricin^cs^ to analyze whether caspase activation is required in the growth-depleted territory or in the neighboring cell populations. DIAP1 represses both DrIcE and Dronc, while the baculovirus protein p35 specifically represses effector caspases DrIce and Dcp-1 and maintains fully active Dronc (reviewed in [25], Figure 3Q). Expression of either DIAP1 or p35 in the same domain as Ricin^cs^ caused a clear autonomous reduction in the number of TUNEL-positive cells (Figure 3J and unpublished data) as well as a clear non-autonomous rescue of BrdU incorporation levels (Figure 3O and unpublished data). A similar non-autonomous rescue in the expression levels of *cycE* and *string* mRNA and in the number of mitotic figures was observed upon p35 expression (Figure 3K, L, P). Similar results were obtained when PTEN or 4E-BP^AA^ were co-expressed with p35 in different domains of the wing disc (Figure 3F, G). We then used FACS and FSC analysis to characterize the cell cycle profile and the size of A cells upon expression of Ricin^cs^ and GFP, or Ricin^cs^ and p35 in the P compartment (with the *en-Gal4* driver, Figure S3). Similar cell cycle profiles and cell sizes were obtained in both genotypes. Comparable results were obtained with the *ci-Gal4* driver (Figure S3). The capacity of p35 expression to rescue the non-autonomous reduction in BrdU, *string*, and *cycE* levels and in mitotic activity caused by *Ricin^cs^* expression, even though Dronc is fully active under these conditions [25], suggests that effector caspases, such as DrIce and Dcp-1, are required in the growth-depleted territory to induce a non-autonomous reduction in cell proliferation rates in the nearby cell populations.

We next analyzed the resulting adult wings when the activity or expression of various elements of the apoptotic machinery was depleted in Ricin^cs^-expressing larvae. The autonomous reduction in tissue size was not rescued and the cell densities were either unaffected or increased when apoptosis was reduced (Figure 3R, S), thereby suggesting that effector caspases do not play a major role in the autonomous reduction in tissue size caused by Ricin^cs^ expression. Surprisingly, the non-autonomous reduction in tissue size was not rescued either (Figure 3R). Nevertheless, and consistent with the role of effector caspases in regulating cell proliferation rates in nearby territories, cell densities were significantly increased in these territories in all the genetic backgrounds tested (Figure 3S). The non-autonomous reduction in cell size is most probably occurring during post-larval stages, since no major change in cell size was observed in imaginal tissues (Figure S2). These results imply that growth and proliferation rates are independently regulated and that the decrease in cell proliferation rates does not play a major role in the observed reduction in tissue size. While effector caspases are required in the growth-depleted territory to induce a non-autonomous reduction in cell proliferation rates in the nearby cell populations, the non-autonomous reduction in tissue size relies on a caspase-independent mechanism.

### A Non-Autonomous Role of dp53 in Regulating Growth and Proliferation Rates

**Figure 4:**
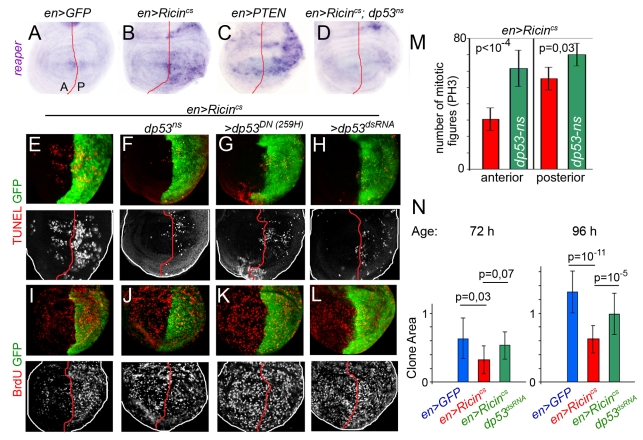
A non-autonomous role of dp53 in regulating proliferation rates. (A–D) Wing discs expressing GFP (A), *Ricin^cs^* (B, D), or *PTEN* (C) with the *en-gal4* driver and labeled to visualize *reaper* mRNA expression. The wing disc shown in (D) is also mutant for dp53 (*dp53^ns^*). (E–M) Wing discs expressing GFP and *Ricin^cs^* with the *en-gal4* driver in different genetic backgrounds (*dp53^ns^* in F, J; co-expressing *dp53^259H^* in G, K; or *dp53^dsRNA^* in H, L) and labeled to visualize GFP, TUNEL-positive cells and BrdU incorporation. (M) Histograms plotting the number of mitotic figures (PH3-positive cells) observed in the A and P compartments of wing discs expressing *Ricin^cs^* in a wild-type (red bars) or a *dp53^ns^* mutant background (green bars). Number of PH3-positive cells: *en> Ricin^cs^*: A = 31±6, P = 58±6; *en> Ricin^cs^*;*dp53^ns^*: A = 64±8, P = 70±7. Ten wing discs were scored per genotype. (N) Histograms plotting the size of clones (in arbitrary units) located in the A compartment of *en-gal4;UAS- Ricin^cs^ UAS-GFP* (blue bars), *en-gal4;UAS- Ricin^cs^ UAS-GFP* (red bars), and *en-gal4; UAS-Ricin^cs^ UAS-dp53^RNAi^*(green bars) wing discs. Clones were generated at the beginning of the Ricin^cs^ induction period and quantified 72 or 96 h later in third instar wing discs. Error bars indicate standard deviation. Size of clones at 72 h: *en>GFP* = 0.67±0.3 (*n* = 28); *en> Ricin^cs^*  = 0.33±0.2 (*n* = 34); *en> Ricin^cs^;p53^RNAi^*  = 0.54±0.2 (*n* = 39). Size of clones at 96 h: *en>GFP* = 1.31±0.3 (*n* = 17); *en> Ricin^cs^*  = 0.63±0.2 (*n* = 15); *en> Ricin^cs^;p53^RNAi^*  = 0.9±0.3 (*n* = 18).

The transcription factor and tumor suppressor p53, a short-lived, non-abundant protein in healthy cells, plays a major role in regulating the response of mammalian cells to stress, in part through the transcriptional activation of genes involved in apoptosis and cell cycle regulation [26]. Impaired TOR signaling, ribosomal biogenesis, and protein translation increase p53 activity [21],[27]. Although the regulation of dp53 in *Drosophila* has not been fully elucidated, the biological function of p53 is well-conserved between flies and mammals [28]. dp53 mediates a variety of stress responses by inducing the expression of the pro-apoptotic gene *reaper* [29],[30]. Interestingly, expression of *reaper* was induced in Ricin^cs^- and in PTEN-expressing cells and this expression depended on the activity of dp53 (Figure 4A-D). We did not find any evidence of increased *dp53* mRNA levels in the transgene-expressing cells (unpublished data), thus suggesting that the activation of dp53 is post-transcriptional. The increase in the number of TUNEL-positive cells caused by expression of Ricin^cs^ was largely rescued by reducing the activity of dp53 in the whole wing disc (in *dp53^ns^* mutant larvae) or in the transgene-expressing domain (co-expressing dp53^DN^ or dp53^dsRNA^; Figure 4E–H). Most interestingly, the effects of Ricin^cs^ on the levels of BrdU incorporation and on the number of mitotic PH3-positive cells were also largely rescued by dp53 depletion (Figure 4I–M). These results indicate that dp53 and the effector caspases are required in the growth-depleted territory to non-autonomously reduce the proliferation rates in neighboring cell populations.

**Figure 5:**
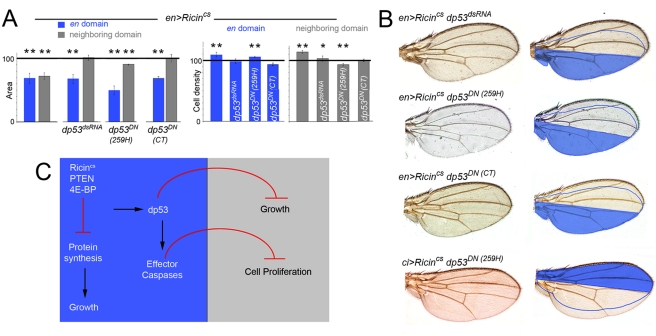
dp53 mediates the coordination of growth between compartments. (A) Histograms plotting the size and cell density values, normalized as a percent of the control GFP-expressing adult wing values, of the *en* domains (blue bars) expressing *Ricin^cs^* alone or together with dp53^dsRNA^, *dp53^CT^*, or *dp53^259H^* and of the neighboring domains not expressing the transgene (gray bars). Error bars indicate standard deviation. Areas were strongly reduced in the transgene-expressing domains but only slightly decreased in the neighboring domains (see also Table S2). Cell densities were significantly changed in those bars labeled by asterisks (see also Table S3, * *p*<0.05 and ** *p*<0.01). (B) Cuticle preparations of adult wings expressing *Ricin^cs^* together with dp53^dsRNA^, *dp53^CT^*, or *dp53^259H^* with the *en-gal4* and *ci-gal4* drivers. The domain of transgene expression is labeled in blue. The expected size of the neighboring domain to give rise to a well-proportioned adult wing is also depicted. (C) Targeted depletion of the insulin pathway or the protein biosynthetic machinery induces the activation of dp53 and the apoptotic machinery. While growth rates and cell cycle length are regulated in a coordinated and non-autonomous manner by the activity of dp53, effector caspases have a unique role, downstream of dp53, in reducing cell proliferation rates in adjacent cell populations.

We next analyzed the resulting adult wings when the activity or expression of *dp53* was depleted in the domains expressing Ricin^cs^. Consistent with the observation that the apoptotic machinery and effector caspases do not play a major role in the autonomous reduction in tissue size caused by expression of these transgenes, the autonomous reduction in tissue size was still observed and the cell densities were either unaffected or increased when *dp53* activity was reduced (Figure 5A). However, and in contrast to the apoptotic machinery and the effector caspases, dp53 exerts a fundamental role in the non-autonomous regulation of tissue size. The non-autonomous reduction in tissue and cell size caused by Ricin^cs^ expression was either weaker or not observed when dp53 activity was depleted in the transgene-expressing domain (Figure 5A), and the resulting adult wings were not well-proportioned and exhibited a clear asymmetric shape with respect to the AP boundary (Figure 5B). In other words, in a situation of reduced dp53 activity in the Ricin^cs^-expressing compartment, the neighboring compartment exhibited a size nearly identical to the one observed in GFP control wings. These results indicate that, upon growth depletion, dp53 exerts a fundamental and non-autonomous role in reducing the size of the adjacent cell populations. In order to address whether growth rates are also regulated in a non-autonomous manner by the activity of dp53, we induced neutral clones of cells at the beginning of the Ricin^cs^ induction period (early second instar) and examined, 72 h and 96 h later, the size of clones located in the A compartment of wing discs expressing GFP, or Ricin^cs^ and GFP, or Ricin^cs^ and dp53^dsRNA^ in the P compartment (with the *en-gal4* driver, Figure 4N). Interestingly, the non-autonomous reduction in clone size caused by Ricin^cs^ expression was largely rescued by co-expression of dp53^dsRNA^ (Figure 4N).

## Discussion

The *Drosophila* wing primordium increases about a thousand-fold in cell mass and cell number during larval development. Even though molecules of the Wnt, TGF-β, and Hedgehog families are well known to organize tissue growth and patterning of this primordium and to play a fundamental role in the generation of an adult wing with a species-specific size, shape, and patterning [31], the molecular mechanisms that contribute to buffering local variations in tissue growth caused by different types of stress and help to generate well-proportioned adult wings under these circumstances remain largely unknown. Here we underscore a new role of *dp53* and the apoptotic machinery in these processes. Depletion of the insulin pathway or the protein biosynthetic machinery in defined territories of the developing wing primordium induces an autonomous reduction in growth rates as well as a non-autonomous decrease of growth and cell proliferation rates in the adjacent cell populations. This non-autonomous response occurs independently of where growth is affected and is functional throughout development. We present evidence that targeted depletion of the insulin pathway or the protein biosynthetic machinery induces the activation of dp53 and consequently the apoptotic machinery. While growth and proliferation rates are regulated in a coordinated and non-autonomous manner by the activity of dp53, effector caspases have a unique role, downstream of dp53, in reducing proliferation rates in adjacent cell populations (Figure 5C). Thus, tissue growth and proliferation rates can be uncoupled and are regulated by two different mechanisms downstream of dp53. These new findings underscore a new role of dp53 and effector caspases in buffering local variations in tissue growth and in maintaining the relative proportions of distinct cell populations within a tissue to give rise to a functional adult structure.

Independent lines of evidence support the view that adjacent cell populations are able to buffer local variations in tissue growth caused by different means, and not only when the activities of the insulin pathway or the protein biosynthetic machinery are compromised. The halteres and wings of *Drosophila* are homologous thoracic appendages, which share common positional information provided by signaling pathways. The activity in the haltere discs of the *Ultrabithorax* (*Ubx*) *Hox* gene establishes the differences between these structures, their different size being an obvious one (reviewed in [32]). In *Contrabithorax* mutant wings in which one compartment is reduced in size due to the transformation to haltere by the ectopic expression of Ubx, the adjacent compartment adjusts its final size and results in a smaller wing territory [33]. Similar non-autonomous effects in tissue size were observed when inducing clones of cells with reduced EGF-Receptor activity [34], upon depletion of the *dMyc* proto-oncogene or over-expression of the *hippo* tumor-suppressor gene (Figure S5). Nevertheless, we cannot rule out the possibility that in other situations in which growth is being challenged, the tissue might not respond as a whole. In these situations, we speculate that dp53 is inactive or not activated. We would like also to point out that the dp53 dependent mechanism described in this work might be functional to buffer local and slight variations in growth rates. Above a certain threshold in the reduction of tissue size, this mechanism might not be sufficient to generate well-proportioned organs.

In vertebrates and invertebrates, p53 and caspase-dependent cell death also play a fundamental role in regeneration (reviewed in [35]) as well as in response to stress or tissue damage, whereby the damaged tissue undergoes extra cell proliferation to compensate for cell loss [23],[36]–[38]. Upon tissue damage in *Drosophila* tissues, a feedback loop mediated by dp53 and the initiator caspase Dronc is required in undifferentiated dying cells to induce cell proliferation in surrounding cells [30]. A specific role of effector caspases has been described in differentiated neurons to induce, upon tissue damage, cell proliferation in surrounding cells [39]. These non-autonomous effects are mediated by the ectopic activation of signaling molecules of the Wnt, TGF-β, and Hedgehog families. In contrast, we did not find any clear change in the expression of Wnt, TGF-β, or Hedgehog in the growth-depleted territory or in the activity of their signaling pathways in adjacent cell populations (Figure S4 and unpublished data). Also, while dp53 and caspases have a role in inducing cell proliferation within the damaged tissue [30], our data indicate that the same molecules reduce growth and proliferation rates in adjacent unaffected cell populations. These observations suggest that different types of signaling molecules induced or activated by dp53 and caspases exert different effects in the damaged tissue and in nearby ones. It is interesting to note in this context that effector caspases have been recently shown to promote wound healing and tissue regeneration in mice by mediating the cleavage of iPLA2 (Calcium-independent Phospholipase A2) to trigger the production of the growth signal Prostaglandin E2 [23]. Whether the non-autonomous reduction in cell proliferation rates is due to the caspase-dependent cleavage and activation of specific signaling molecules or whether dying cells release a variety of signaling molecules as a consequence of cell demolition [40] that could have a general role in the reduction of cell proliferation rates remains to be solved. Similarly, the signaling molecules that mediate the non-autonomous role of dp53 in regulating tissue growth remain to be identified.

Finally, we would like to highlight the central role of p53 in tissue homeostasis and stress response. While the role of p53 in regulating the response of mammalian cells to stress through the transcriptional activation of genes involved in apoptosis and cell cycle arrest is cell-autonomous [41], we speculate that the non-autonomous role of dp53 defined in this study also makes a major contribution to stress response and tissue homeostasis. Upon tissue damage, neighboring populations of healthy cells might reduce their growth and proliferation rates until damaged cells have been recovered. The combined autonomous and non-autonomous activities of p53 might be fundamental in tissue homeostasis.

## Materials and Methods

### *Drosophila* Strains

The *Drosophila* strains include: *UAS-Ricin^cs^* ([10], a cold sensitive version of the Ricin toxin A Chain), *UAS-4EBP^AA^* (a 4E-BP constitutively active form with two threonine-to-alanine substitutions (T37A, T46A) [12]), *UAS-PTEN* [13]; *sal^PE^-Gal4* [16]; *UAS–dp53^RNAi^* (ID 10692, VDRC); and *dp53^ns^* (a knock out of *dp53* created by ends-in homologous recombination, Flybase). Other stocks are described in Flybase or Text S1.

### Immunohistochemistry and In Situ Hybridization

Mouse anti-BrdU (Developmental Studies Hybridoma Bank); rabbit anti-PH3 (Upstate Biotechnology); rabbit anti-GFP (Abcam); rabbit anti-cleaved-Caspase 3 (Asp175, Cell Signaling Technology). Other antibodies are described in Text S1. Secondary antibodies were obtained from Molecular Probes. TUNEL analysis, BrdU staining, and in situ hybridization were performed as described in [20]. A Digoxigenin-RNA Labeling kit (Roche) was used to synthesise probes of *string*, *cycE*, and *reaper*.

### Quantification of Tissue and Cell Size in Adult Wings

Wing size and size of the A and P compartments were measured using the Image J Software (NIH, USA). Cell density was measured as the number of hairs (each wing cell differentiates a hair) per defined area. Two conserved regions between veins L4 and L5 (P compartment) and veins L2 and L3 (A compartment) in both dorsal and ventral surfaces of the wings were used to measure cell densities. The final area and cell density values were normalized as a percent of the control Gal4-driver; UAS-GFP values. At least 10 adult wings per genotype were scored. Only adult males were scored. The average values and the corresponding standard deviations were calculated and *t* test analysis was carried out.

### Quantification of Tissue Growth in Developing Wing Discs

*en-Gal4/SM6a-TM6b/UAS-Ricin^cs^* or *en-Gal4, UAS-GFP* females were crossed with *w^1118^/Y* males and allowed to lay eggs for 5 h at 18°C. After 6 days at 18°C, the resulting larvae were transferred to 29°C. Wing discs were dissected after different time points of *Ricin^cs^* induction and tissue size was measured from confocal images by means of Image J software (NIH, USA). At least 10 wing discs were used per time point. Average values and the corresponding standard deviations were calculated and *t* test analysis was carried out. Note that, consistent with the temperature sensitivity of Ricin^cs^, *en-gal4;UAS-Ricin^cs^* adult wings grown at the restrictive temperature (18°C) showed a size nearly identical to the one of *en-gal4;UAS-GFP* adult wings (Figure S1),

### Growth Rate Measurements by Clonal Analysis

*hs-FLP*, *ubi-GFP*, *FRT19* or *hs-FLP*, *ubi-GFP*, *FRT19; +/+; UAS-p53^RNAi^* females were crossed with control *FRT19/Y* and experimental *FRT19/Y; en-Gal4/SM6a-TM6b/UAS-Ricin^cs^* males and allowed to lay eggs for 5 h at 18°C. After 6 days at 18°C, the resulting larvae were heat-shocked at 37°C for 30 min and then transferred to 29°C. Wing discs were dissected 72 h and 96 h after clone induction. The size of clones was quantified from confocal images with Image J software (NIH, USA). At least 15 clones were quantified per time point. Average values and the corresponding standard deviations were calculated and *t* test analysis was carried out.

### Flow Cytometry Analysis

Wing discs of larvae carrying different UAS transgenes and the *en-Gal4* or *ci-gal4* drivers were dissected and cells were dissociated according to the protocol described in [42]. Hoescht and GFP fluorescence were determined by flow cytometry using a MoFlo flow cytometer (DakoCytomation, Fort Collins, CO). Excitation of the sample was carried out using a Coherent Enterprise II argon-ion laser. Excitation with the blue line of the laser (488 nm) permits the acquisition of forward-scatter (FS), side-scatter (SS), and green (530 nm) fluorescence from GFP. UV emission (40 mW) was used to excite Hoescht blue fluorescence (450 nm). Doublets were discriminated using an integral/peak dotplot of Hoechst fluorescence. Optical alignment was based on optimized signal from 10 µm fluorescent beads (Flowcheck, Coulter Corporation, Miami, FL). DNA analysis (Ploidy analysis) on single fluorescence histograms was done using Multicycle software (Phoenix Flow Systems, San Diego, CA).

### Analysis of Developmental Delay

Forty hatched first-instar larvae were sorted from egg laying plates (egg laying period of 5 h), transferred to fresh tubes, and kept at 18°C. Then, animals of the different genotypes were transfer to 29°C from early second instar and the number of pupae was counted daily. Results were expressed as percentage of the individuals from each genotype that attained the pupal stage.

## Supporting Information

Figure S1 **Adjacent cell populations attain a final size in a coordinated manner.** (A) Cuticle preparations of adult wings expressing GFP and Ricin^cs^ under the control of different Gal4 drivers. The reduction in wing size caused by Ricin^cs^ expression when compared to GFP-expressing wings is shown. At least 10 wings per genotype were measured. Only adult males were scored. Expression domains of gal4 drivers are shown in wing discs expressing the *UAS-lacZ* transgene and stained for β-Galactosidase activity (blue, A). Wing areas: *hh>GFP* = 90±3; *ptc>Ricin^cs^*  = 78±4; *dpp>GFP* = 75±4; *IJ3>Ricin^cs^*  = 90±5; hth*>GFP* = 90±4; *tsh> Ricin^cs^*  = 80±4. Localized transgene expression induced a significant reduction in adult wing size when compared to GFP-expressing wings raised in the same conditions (*p*<10^−4^). (B, C) Histograms plotting the size (B) and cell density values (C), normalized as a percent of the control GFP-expressing wing values, of the *hh*, *ptc*, *and dpp* domains expressing *Ricin^cs^* (blue bars) and of the neighboring domains not expressing the transgene (grey bars). Error bars indicate standard deviation. Horizontal line shows the size or cell density values of the normalized control GFP-expressing wing values. At least 10 wings were analyzed per genotype. Only adult males were scored. Areas were significantly reduced in the transgene-expressing and non-expressing domains (*p*<10^−3^; see also Table S2). Cell densities were significantly increased in those bars labeled by asterisks (* *p*<0.05; ** *p*<0.01; see also Table S3). (D) Time-lapse experiments showing the coordinated reduction of tissue size all throughout development. Animals expressing GFP or Ricin^cs^ in the *ci-gal4*-expressing domain (anterior compartment) were transferred to 29°C at different developmental time points until adult eclosion. Ricin^cs^ expression induced a reduction in total tissue size. Area (control): 100±2; Area (early L2-adult): 66±4; Area (early L3-adult): 80±5; Area (mid L3-adult): 86±4; Area (early pupa-adult): 91±5. The total area was significantly reduced when compared to GFP control wings raised at 29°C (*p*<10^−3^). (E) Histograms plotting the wing size, normalized as a percent of the control GFP-expressing wing values, of the *en-gal4; UAS-Ricin^cs^* flies grown at the restrictive temperature (18°C). The *Ricin^cs^*-expressing domain (blue bar) and the neighboring domain not expressing the transgene (grey bar) are shown. Error bars indicate standard deviation. Horizontal line shows the size of the normalized control GFP-expressing wing values. Wing areas: *en>GFP*: *A* = 100±4.8, P = 100±4.1; *en> Ricin^cs^*: *A* = 102.6±3.6, P = 102.9±4.8.
(1.56 MB TIF)

Figure S2 **Targeted depletion of growth induces a non-autonomous reduction in growth and proliferation rates in adjacent cell populations.** (A, B) *Ricin^cs^*-expressing animals extended their larval period for about 24–36 h before entering into pupariation. Animals expressing GFP (blue lines) or Ricin^CS^ (red lines) with en-Gal4 (A) or vestigial-Gal4 (B) were transferred to 29°C from early second instar and the number of pupae were quantified every 24 h. Results are expressed as percentage of the individuals from each genotype that attained the pupal stage. (C, D) Fluorescence associated cell sorter (FACS) and forward scatter (FSC) analysis of the non GFP cells. (C) *en-gal4;UAS-*GFP (blue line) and *en-gal4;UAS-Ricin^cs^* (red line). (D) *ci-gal4;UAS-*GFP (blue line) and *ci-gal4;UAS-Ricin^cs^* (red line). Percentage of cells in G1, G2, and S are indicated bellow each graphic. (E–G) *ptc-gal4;UAS-Ricin^cs^* (E), *ci-gal4; UAS-Ricin^cs^* (F), *nub-gal4;UAS-Ricin^cs^* (G) wing discs labeled to visualize BrdU incorporation (red or white) and GFP (green).
(1.28 MB TIF)

Figure S3 **A non-autonomous role of effector Caspases in regulating proliferation rates.** (A, B) Fluorescence associated cell sorter (FACS) and forward scatter (FSC) analysis of the non GFP cells. (A) *en-gal4;UAS-Ricin^cs^* (red line) and *en-gal4;UAS-Ricin^cs^*p35 (green line). (B) *ci-gal4;UAS-Ricin^cs^* (red line) and *ci-gal4;UAS-Ricin^cs^*p35 (green line). Percentage of cells in G1, G2, and S are indicated bellow each graphic.
(0.22 MB TIF)

Figure S4 **Activity levels of the main organizing signaling pathways upon Ricin^cs^ expression**. (A–H) *Wild-type* wing discs (A–D) or wing discs expressing Ricin^cs^ and GFP (green) in the *engrailed* (*en*, E, F) or *apterous* (*ap*, G, H) domains and labeled to visualize Wingless (Wg, A, E), Senseless (Sens, B, F), Spalt (C, G), and Patched (Ptc, D, H) protein expression in red or white. Sens, Spalt, Wg, and Ptc are molecular readouts of the activity levels of the Wg, Dpp, Notch, and Hh signaling pathways in the developing wing.
(2.71 MB TIF)

Figure S5 **Non-autonomous reduction of tissue size and proliferation rates caused by depletion of** ***dMyc*** **or overexpression of** ***hippo*** **.** (A) Cuticle preparation of an adult wing in which the domain of expression of the *sal^PE^-Gal4* driver is depicted in red and the neighboring domains in blue. AWM, anterior wing margin; PWM, posterior wing margin; L2 and L5, longitudinal veins. (B) Histogram plotting the tissue size, normalized as a percent of the control GFP-expressing wing values, of *sal^PE^ > dMyc^dsRNA^* adult wings (dark blue bar), sal^PE^ domains expressing *dMyc^dsRNA^* (red bar), and adjacent domains not expressing the transgene (light blue bars). Error bars indicate standard deviation. The horizontal line shows the size value of the normalized control GFP-expressing wings. Areas were significantly reduced in the transgene-expressing and non-expressing domains (see also Tables S1 and S2, ** *p*<0.01). (C–D) *W*ing discs expressing *dMyc^dsRNA^* and GFP (green) in the *engrailed* (*en*) domain and labeled to visualize dMyc (blue or white, C) and BrdU incorporation (white). Note reduced incorporation of BrdU in the transgene non-expressing compartment. (E) Histogram plotting the tissue size, normalized as a percent of the control GFP-expressing wing values, of the *en* domain expressing *dMyc^dsRNA^* (blue bar) and the adjacent compartment not expressing the transgene (white bars). Error bars indicate standard deviation. The horizontal line shows the size value of the normalized control GFP-expressing wings. Areas were significantly reduced in the transgene-expressing and non-expressing domains (see also Table S2, ** *p*<0.01). (F) *W*ing disc expressing *hippo* and GFP (green) in the *ci* domain and labeled to BrdU incorporation (red or white). Note reduced incorporation of BrdU in the transgene non-expressing compartment. (G) Histogram plotting the tissue size, normalized as a percent of the control GFP-expressing wing values, of the *en* domain expressing *hippo* (blue bar) and the adjacent compartment not expressing the transgene (white bars). Error bars indicate standard deviation. The horizontal line shows the size value of the normalized control GFP-expressing wings. Areas were significantly reduced in the transgene-expressing and non-expressing domains (see also Table S2, * *p*<0.05, ** *p*<0.01).
(0.93 MB TIF)

Table S1 **Tissue size values of whole wings (total wing area) expressing the** ***Ricin^cs^*** **,** ***PTEN*** **, or** ***4E-BP^AA^*** **transgenes in different domains measured as a ratio (in percentage) with respect to control wings expressing GFP in the same domains (underlined).** These values correspond to the average of 10 adult wings with their corresponding standard deviations. A *t* test was carried out to calculate the *p* value as a measurement of the statistical significance of the difference between transgene-expressing and GFP-expressing wings.
(0.08 MB DOC)

Table S2 **Tissue size values of compartments or wing territories expressing and not expressing undergrowth promoting transgenes.** (A)Tissue size values of compartments expressing and not expressing the *Ricin^cs^*, *PTEN*, or *4E-BP^AA^* transgenes and measured as a ratio (in percentage) with respect to control wings expressing GFP in the same domains (underlined). (B) Tissue size values of wing territories expressing and not expressing a *dMyc^dsRNA^*, transgene and measured as a ratio (in percentage) with respect to control wings expressing GFP in the same domains (underlined). The transgene was expressed in the spalt domain (between longitudinal veins L2 and L5). The areas of the following neighboring domains were also measured: (1) AWM-L2: area between the anterior wing margin and longitudinal vein L2, and (2) L5-PWM: area between longitudinal vein L5 and the posterior wing margin. These values correspond to the average of 10 adult wings with their corresponding standard deviations. A *t* test was carried out to calculate the *p* value as a measurement of the statistical significance of the difference between transgene-expressing and GFP-expressing wings.
(0.08 MB DOC)

Table S3 **Cell density values of compartments expressing and not expressing the Ricincs, PTEN, or 4E-BPAA transgenes and measured as a ratio (in percentage) with respect to control wings expressing GFP in the same domains (underlined).** These values correspond to the average of 10 adult wings with their corresponding standard deviations. A *t* test was carried out to calculate the *p* value as a measurement of the statistical significance of the difference between transgene-expressing and GFP-expressing wings.
(0.06 MB DOC)

Table S4 **Tissue size values of** ***Ricin^cs^*** **-expressing and non-expressing compartments measured as a ratio (in percentage) with respect to control wings expressing GFP in the same domain (underlined).** These values correspond to the average of 10 adult wings with their corresponding standard deviations. A *t* test was carried out to calculate the *p* value as a measurement of the statistical significance of the difference between transgene-expressing and GFP-expressing wings. Larvae were grown at 18°C and switched to 29°C at different times of development until adulthood.
(0.04 MB DOC)

Text S1 **Supporting Materials and Methods.**
(0.04 MB DOC)

## Abbreviations

4E-BP: eukaryotic translation initiation factor 4E binding protein
AP: anterior-posterior
BrdU: bromodeoxyuridine
*cycE*: *cyclin E*
DIAP1: Drosophila inhibitor of apoptosis 1
dp53: *Drosophila* p53
FACS: fluorescence-associated cell sorter
FSC: forward scatter
IAP: inhibitor-of-apoptosis protein
IGF: insulin-like growth factor
PH3: phosphorylated histone H3
PTEN: phosphatase and tensin homolog
*stg*: *string*
TOR: Target of Rapamycin
