# Supplementary figures and images for: A dp53-Dependent Mechanism Involved in Coordinating Tissue Growth in Drosophila
Source: PLoS Biol. 2010 Dec 14;8(12):e1000566. doi: 10.1371/journal.pbio.1000566 (PMC3001892; doi:10.1371/journal.pbio.1000566)

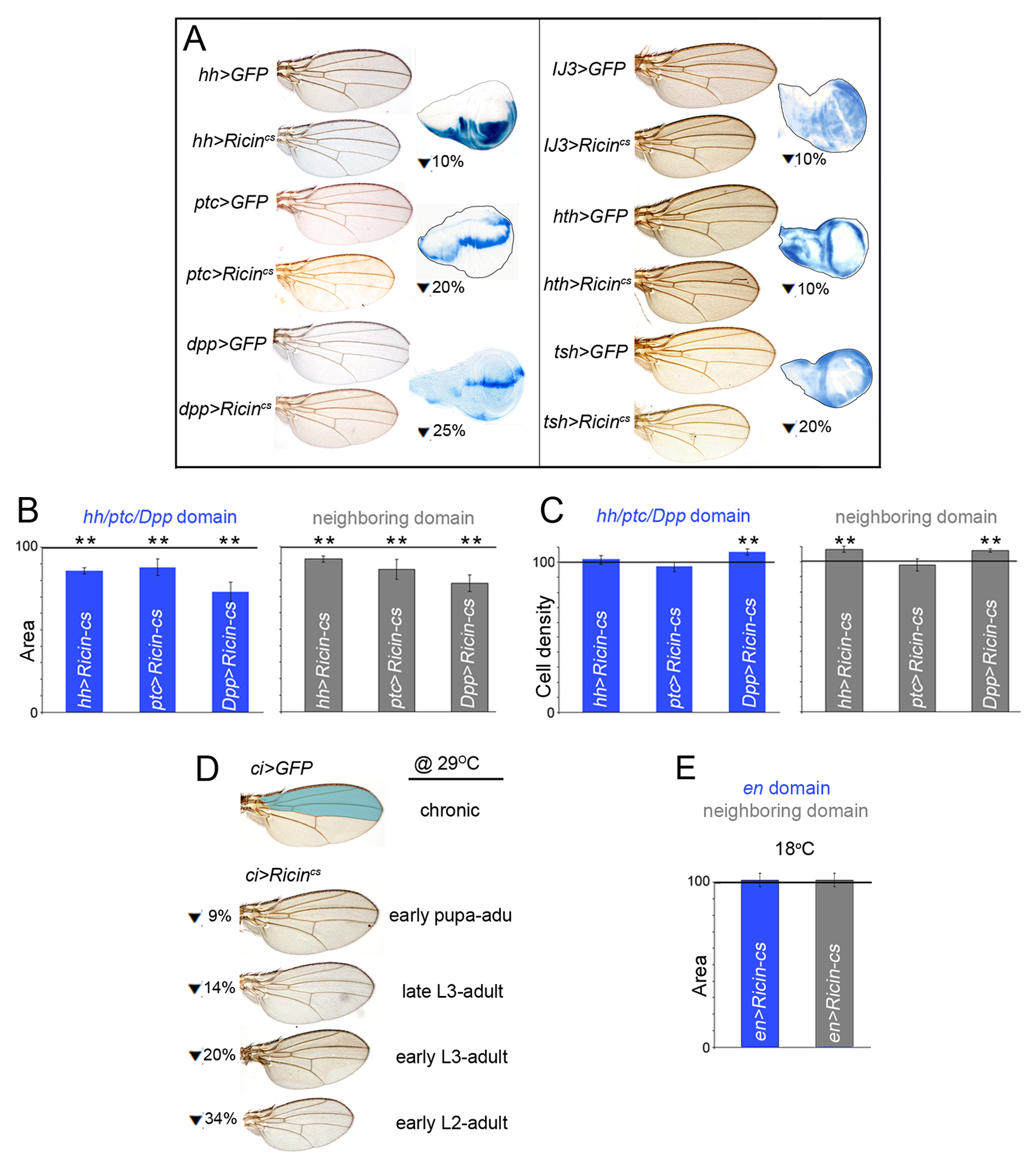

Supplement: Figure S1 — Adjacent cell populations attain a final size in a coordinated manner. (A) Cuticle preparations of adult wings expressing GFP and Ricincs under the control of different Gal4 drivers. The reduction in wing size caused by Ricincs expression when compared to GFP-expressing wings is shown. At least 10 wings per genotype were measured. Only adult males were scored. Expression domains of gal4 drivers are shown in wing discs expressing the UAS-lacZ transgene and stained for β-Galactosidase activity (blue, A). Wing areas: hh>GFP = 90±3; ptc>Ricincs = 78±4; dpp>GFP = 75±4; IJ3>Ricincs = 90±5; hth>GFP = 90±4; tsh> Ricincs = 80±4. Localized transgene expression induced a significant reduction in adult wing size when compared to GFP-expressing wings raised in the same conditions (p<10−4). (B, C) Histograms plotting the size (B) and cell density values (C), normalized as a percent of the control GFP-expressing wing values, of the hh, ptc, and dpp domains expressing Ricincs (blue bars) and of the neighboring domains not expressing the transgene (grey bars). Error bars indicate standard deviation. Horizontal line shows the size or cell density values of the normalized control GFP-expressing wing values. At least 10 wings were analyzed per genotype. Only adult males were scored. Areas were significantly reduced in the transgene-expressing and non-expressing domains (p<10−3; see also Table S2). Cell densities were significantly increased in those bars labeled by asterisks (* p<0.05; ** p<0.01; see also Table S3). (D) Time-lapse experiments showing the coordinated reduction of tissue size all throughout development. Animals expressing GFP or Ricincs in the ci-gal4-expressing domain (anterior compartment) were transferred to 29°C at different developmental time points until adult eclosion. Ricincs expression induced a reduction in total tissue size. Area (control): 100±2; Area (early L2-adult): 66±4; Area (early L3-adult): 80±5; Area (mid L3-adult): 86±4; Area (early pupa-adult): 91±5 [file pbio.1000566.s001.tif]

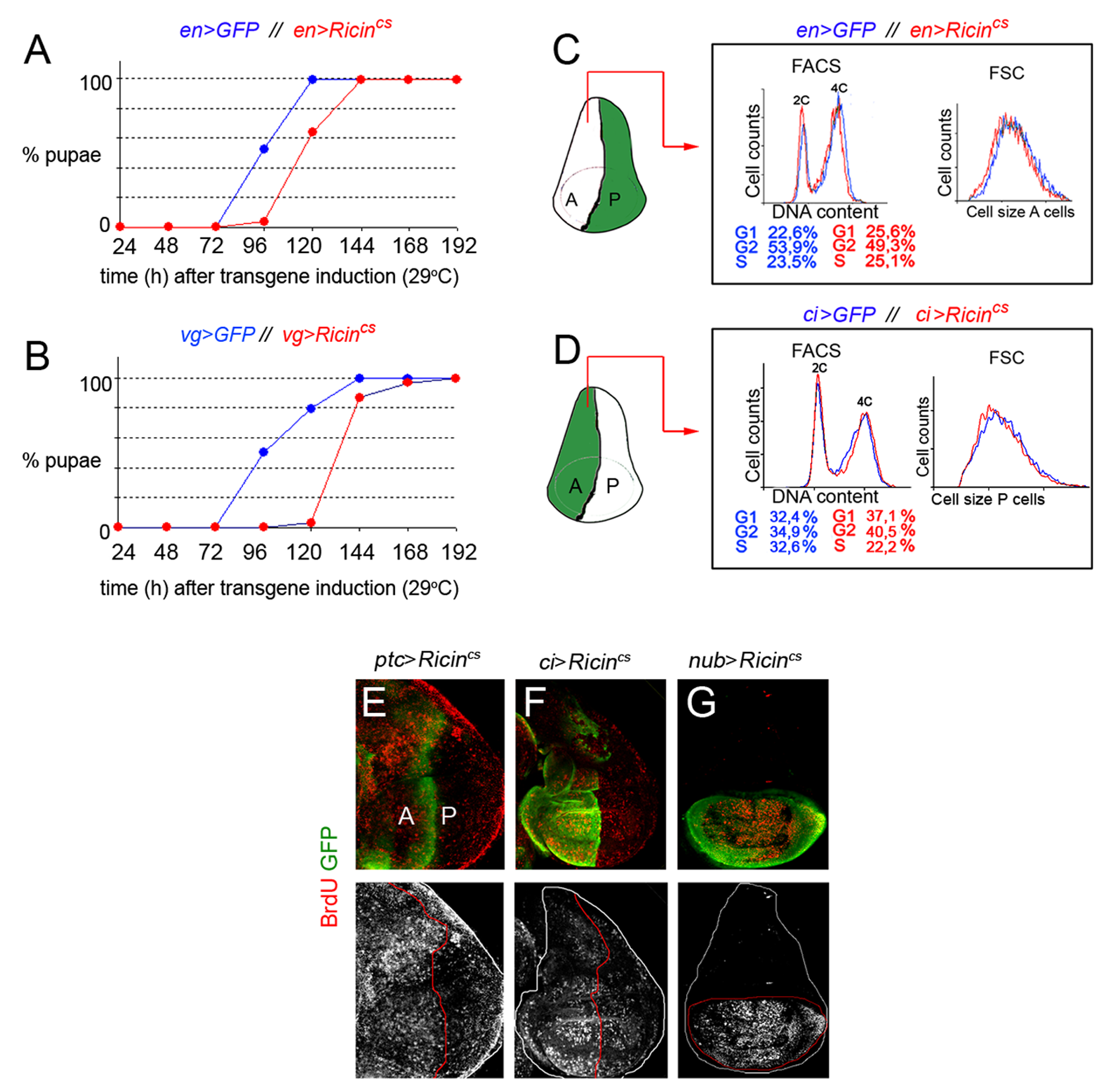

Supplement: Figure S2 — Targeted depletion of growth induces a non-autonomous reduction in growth and proliferation rates in adjacent cell populations. (A, B) Ricincs-expressing animals extended their larval period for about 24–36 h before entering into pupariation. Animals expressing GFP (blue lines) or RicinCS (red lines) with en-Gal4 (A) or vestigial-Gal4 (B) were transferred to 29°C from early second instar and the number of pupae were quantified every 24 h. Results are expressed as percentage of the individuals from each genotype that attained the pupal stage. (C, D) Fluorescence associated cell sorter (FACS) and forward scatter (FSC) analysis of the non GFP cells. (C) en-gal4;UAS-GFP (blue line) and en-gal4;UAS-Ricincs (red line). (D) ci-gal4;UAS-GFP (blue line) and ci-gal4;UAS-Ricincs (red line). Percentage of cells in G1, G2, and S are indicated bellow each graphic. (E–G) ptc-gal4;UAS-Ricincs (E), ci-gal4; UAS-Ricincs (F), nub-gal4;UAS-Ricincs (G) wing discs labeled to visualize BrdU incorporation (red or white) and GFP (green). (1.28 MB TIF) [file pbio.1000566.s002.tif]

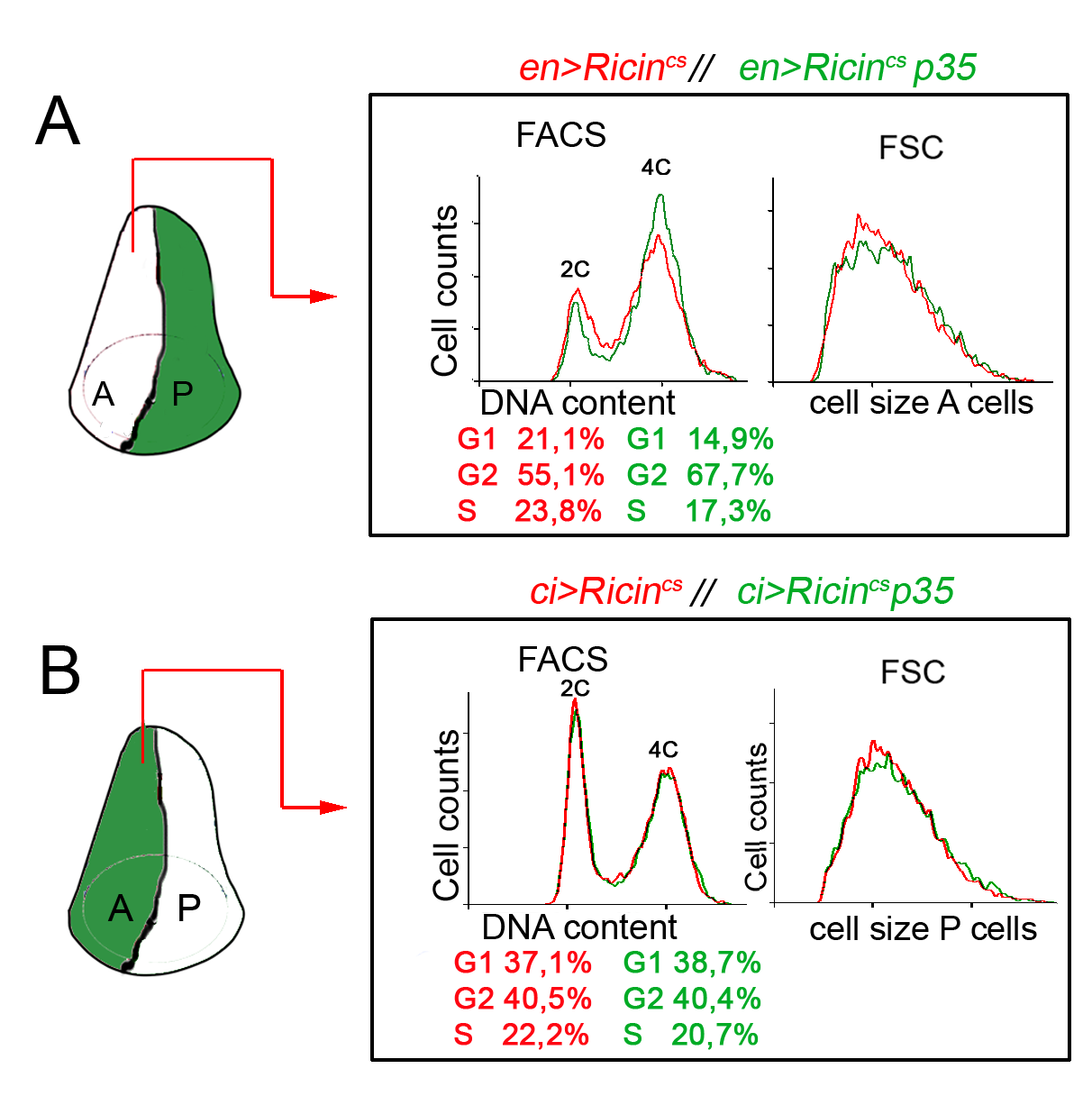

Supplement: Figure S3 — A non-autonomous role of effector Caspases in regulating proliferation rates. (A, B) Fluorescence associated cell sorter (FACS) and forward scatter (FSC) analysis of the non GFP cells. (A) en-gal4;UAS-Ricincs (red line) and en-gal4;UAS-Ricincsp35 (green line). (B) ci-gal4;UAS-Ricincs (red line) and ci-gal4;UAS-Ricincsp35 (green line). Percentage of cells in G1, G2, and S are indicated bellow each graphic. (0.22 MB TIF) [file pbio.1000566.s003.tif]

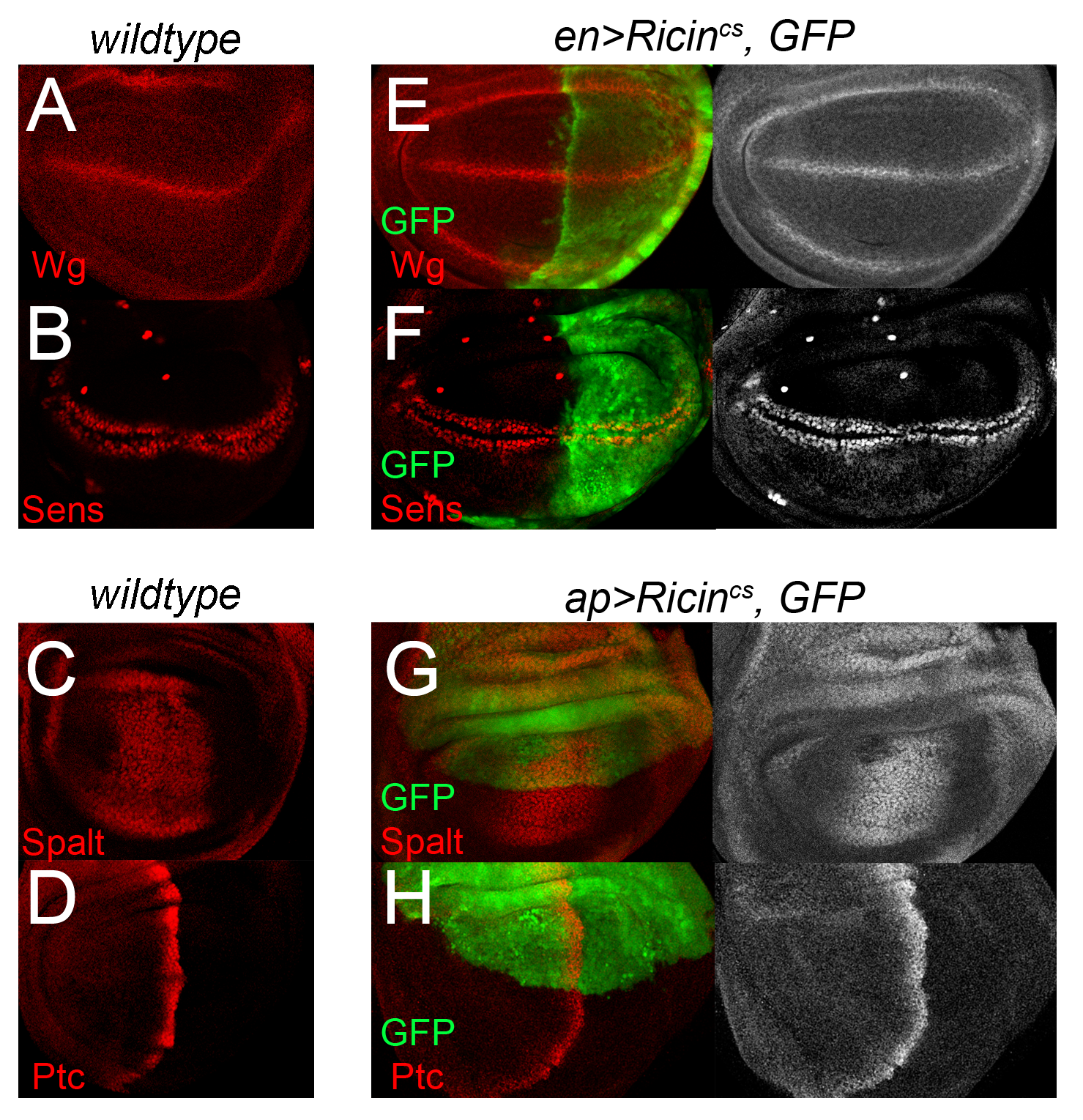

Supplement: Figure S4 — Activity levels of the main organizing signaling pathways upon Ricincs expression. (A–H) Wild-type wing discs (A–D) or wing discs expressing Ricincs and GFP (green) in the engrailed (en, E, F) or apterous (ap, G, H) domains and labeled to visualize Wingless (Wg, A, E), Senseless (Sens, B, F), Spalt (C, G), and Patched (Ptc, D, H) protein expression in red or white. Sens, Spalt, Wg, and Ptc are molecular readouts of the activity levels of the Wg, Dpp, Notch, and Hh signaling pathways in the developing wing. (2.71 MB TIF) [file pbio.1000566.s004.tif]

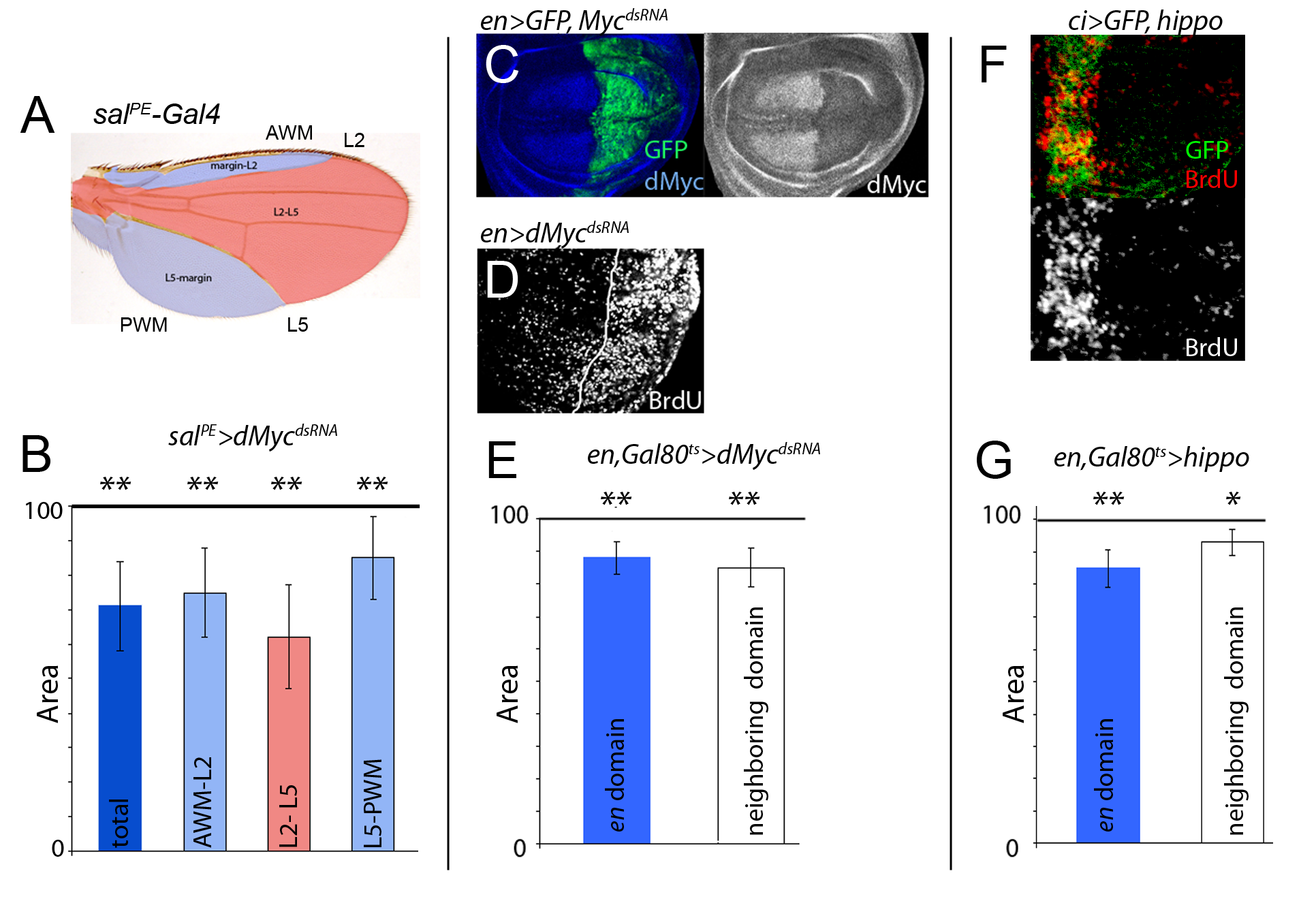

Supplement: Figure S5 — Non-autonomous reduction of tissue size and proliferation rates caused by depletion of dMyc or overexpression of hippo . (A) Cuticle preparation of an adult wing in which the domain of expression of the salPE-Gal4 driver is depicted in red and the neighboring domains in blue. AWM, anterior wing margin; PWM, posterior wing margin; L2 and L5, longitudinal veins. (B) Histogram plotting the tissue size, normalized as a percent of the control GFP-expressing wing values, of salPE > dMycdsRNA adult wings (dark blue bar), salPE domains expressing dMycdsRNA (red bar), and adjacent domains not expressing the transgene (light blue bars). Error bars indicate standard deviation. The horizontal line shows the size value of the normalized control GFP-expressing wings. Areas were significantly reduced in the transgene-expressing and non-expressing domains (see also Tables S1 and S2, ** p<0.01). (C–D) Wing discs expressing dMycdsRNA and GFP (green) in the engrailed (en) domain and labeled to visualize dMyc (blue or white, C) and BrdU incorporation (white). Note reduced incorporation of BrdU in the transgene non-expressing compartment. (E) Histogram plotting the tissue size, normalized as a percent of the control GFP-expressing wing values, of the en domain expressing dMycdsRNA (blue bar) and the adjacent compartment not expressing the transgene (white bars). Error bars indicate standard deviation. The horizontal line shows the size value of the normalized control GFP-expressing wings. Areas were significantly reduced in the transgene-expressing and non-expressing domains (see also Table S2, ** p<0.01). (F) Wing disc expressing hippo and GFP (green) in the ci domain and labeled to BrdU incorporation (red or white). Note reduced incorporation of BrdU in the transgene non-expressing compartment. (G) Histogram plotting the tissue size, normalized as a percent of the control GFP-expressing wing values, of the en domain expressing hippo (blue bar) and the adjacent compartment not expressi [file pbio.1000566.s005.tif]
