# Supplementary material for: A dp53-Dependent Mechanism Involved in Coordinating Tissue Growth in Drosophila
Source: PLoS Biol. 2010 Dec 14;8(12):e1000566. doi: 10.1371/journal.pbio.1000566 (PMC3001892; doi:10.1371/journal.pbio.1000566)
Supplement: Table S1 — Tissue size values of whole wings (total wing area) expressing the Ricincs , PTEN , or 4E-BPAA transgenes in different domains measured as a ratio (in percentage) with respect to control wings expressing GFP in the same domains (underlined). These values correspond to the average of 10 adult wings with their corresponding standard deviations. A t test was carried out to calculate the p value as a measurement of the statistical significance of the difference between transgene-expressing and GFP-expressing wings. (0.08 MB DOC) [file pbio.1000566.s006.doc]

Table S1

| **Total Wing Area (% of controls)** | | | | |
| --- | --- | --- | --- | --- |
| **Genotypes** | | **Total** | | **p-value** |
| *en-G4>GFP* | | 100 | ± 3.7 | - |
| *en-G4>Ricin^CS^* | | 78 | ± 7 | 10^-6^ |
| *en-G4>PTEN* | | lethal | - | - |
| *en-G4>4E-BP^AA^* | | 79 | ± 1.8 | 10^-14^ |
| *ci-G4>GFP* | | 100 | ± 2.3 | - |
| *ci-G4> Ricin^CS^* | | 65 | ± 4 | 10^-15^ |
| *ci-G4>PTEN* | | 78.8 | ± 3 | 10^-11^ |
| *dpp-G4>GFP* | | 100 | ± 4.9 | - |
| *dpp-G4> Ricin^CS^* | | 75 | ± 4.2 | 10^-8^ |
| *patched-G4>GFP* | | 100 | ± 4.6 | - |
| *patched-G4> Ricin^CS^* | | 87 | ± 4.4 | 10^-4^ |
| *hh-G4>GFP* | | 100 | ± 1.6 | - |
| *hh-G4> Ricin^CS^* | | 89.9 | ± 2.7 | 10^-5^ |
| *ap-G4>GFP* | | 100 | ± 2.6 | - |
| *ap-G4> Ricin^CS^* | | 78.47 | ± 2.6 | 10^-5^ |
| *brk-G4>GFP* | | 100 | ± 2.3 | - |
| *brk-G4> Ricin^CS^* | | 85.8 | ± 5.8 | 10^-5^ |
| *C96-G4>GFP* | | 100 | ± 1.4 | - |
| *C96-G4>PTEN* | | 93.8 | ± 2.5 | 10^-4^ |
| *IJ3-G4>GFP* | | 100 | ± 1.3 | - |
| *IJ3-G4> Ricin^CS^* | | 89.5 | ± 5 | 10^-4^ |
| *hth-G4>GFP* | | 100 | ± 3.2 | - |
| *hth-G4> Ricin^CS^* | | 90.8 | ± 4 | 10^-3^ |
| *tsh-G4>GFP* | | 100 | ± 3.2 | - |
| *tsh-G4> Ricin^CS^* | | 79.4 | ± 4 | 10^-9^ |
| *spalt^PE^-G4>GFP* | | 100 | ± 3.9 | - |
| *spalt^PE^-G4>PTEN* | | 82.3 | ± 2.1 | 10^-9^ |
| *en-G4, Gal80^ts^>GFP@29^o^C* | | 100 | ± 4.3 | - |
| *en-G4, Gal80^ts^ >dMyc^dsRNA^@29^o^C* | | 87 | ± 3.1 | 10^-3^ |
| *en-G4, Gal80^ts^>GFP@25^o^C* | | 100 | ± 1.7 | - |
| *en-G4, Gal80^ts^ >hippo@25^o^C* | | 87 | ± 4.6 | 10^-5^ |
| *spalt^PE^-G4>GFP@30^o^C* | | 100 | ± 1.9 | - |
| *spalt^PE^-G4>dMyc^dsRNA^@30^o^C* | | 71 | ± 1.3 | 10^-6^ |
| *en-G4> Ricin^CS^* | *>p35* | 85.2 | ± 3.5 | 10^-8^ |
|  | *Df(H99)/+* | 90.4 | ± 3.9 | 10^-4^ |
|  | *>Diap1* | 85.6 | ± 1.8 | 10^-7^ |
|  | *dronc^L29^/+* | 73.8 | ± 9.3 | 10^-8^ |
|  | *>dp53^DN(CT))^* | 85% | ± 8.2 | 10^-3^ |
|  | *>dp53^DN(259H)^* | 67.9 | ± 3.9 | 10^-13^ |
|  | *>dp53^dsRNA^* | 83.7 | ± 4.3 | 10^-8^ |
|  | *dp53^ns^* | lethal | - | - |

###### Tissue size values of whole wings (total wing area) expressing the *Ricin^cs^*, *PTEN* or *4E-BP^AA^* transgenes in different domains measured as a ratio (in percentage) with respect to control wings expressing GFP in the same domains (underlined). These values correspond to the average of 10 adult wings with their corresponding standard deviations. A t-test was carried out to calculate the p value as a measurement of the statistical significance of the difference between transgene expressing and GFP expressing wings.
