# Supplementary material for: A dp53-Dependent Mechanism Involved in Coordinating Tissue Growth in Drosophila
Source: PLoS Biol. 2010 Dec 14;8(12):e1000566. doi: 10.1371/journal.pbio.1000566 (PMC3001892; doi:10.1371/journal.pbio.1000566)
Supplement: Table S2 — Tissue size values of compartments or wing territories expressing and not expressing undergrowth promoting transgenes. (A)Tissue size values of compartments expressing and not expressing the Ricincs, PTEN, or 4E-BPAA transgenes and measured as a ratio (in percentage) with respect to control wings expressing GFP in the same domains (underlined). (B) Tissue size values of wing territories expressing and not expressing a dMycdsRNA, transgene and measured as a ratio (in percentage) with respect to control wings expressing GFP in the same domains (underlined). The transgene was expressed in the spalt domain (between longitudinal veins L2 and L5). The areas of the following neighboring domains were also measured: (1) AWM-L2: area between the anterior wing margin and longitudinal vein L2, and (2) L5-PWM: area between longitudinal vein L5 and the posterior wing margin. These values correspond to the average of 10 adult wings with their corresponding standard deviations. A t test was carried out to calculate the p value as a measurement of the statistical significance of the difference between transgene-expressing and GFP-expressing wings. (0.08 MB DOC) [file pbio.1000566.s007.doc]

**Table S2A**

| **Compartment Area (% of controls)** | | | | | | | | |
| --- | --- | --- | --- | --- | --- | --- | --- | --- |
| **Genotypes** | | **Transgene expressing compartment** | | **p-value** | | **Transgene non-expressing compartment** | | **p-value** |
| *en-G4>GFP* | | 100 | ± 3.6 | - | | 100 | ± 2.8 | - |
| *en-G4> Ricin^CS^* | | 72 | ± 8 | 10^-7^ | | 86 | ± 5 | 10^-4^ |
| *en-G4>PTEN* | | Lethal | - | - | | - | - | - |
| *en-G4>4EBP* | | 77 | ± 2 | 10^-9^ | | 89 | ± 3 | 10^-5^ |
| *en-G4, Gal80^ts^>GFP@29 ^o^C* | | 100 | ± 5.6 | - | | 100 | ± 4.8 | - |
| *en-G4, Gal80^ts^ >dMyc^dsRNA^@29 ^o^C* | | 88 | ± 5.2 | 0.003 | | 85 | ± 6.2 | 10^-3^ |
| *en-G4, Gal80^ts^>GFP@25 ^o^C* | | 100 | ± 6.2 | - | | 100 | ± 4.1 | - |
| *en-G4, Gal80^ts^ >hippo@25 ^o^C* | | 85 | ± 8.2 | 0.001 | | 93 | ± 6.5 | 0.019 |
| *ci-G4>GFP* | | 100 | ± 2.5 | - | | 100 | ± 4.3 | - |
| *ci-G4> Ricin^CS^* | | 63 | ± 5.1 | 10^-10^ | | 68 | ± 4.5 | 10^-12^ |
| *ci-G4>PTEN* | | 67 | ± 5.5 | 10^-10^ | | 86 | ± 7.8 | 10^-3^ |
| *dpp-G4>GFP* | | 100 | ± 4.2 | - | | 100 | ± 6.1 | - |
| dpp-*G4> Ricin^CS^* | | 73 | ± 6 | 10^-8^ | | 78 | ± 5.1 | 10^-6^ |
| *ptc-G4>GFP* | | 100 | ± 6.3 | - | | 100 | ± 4.7 | - |
| *ptc-G4> Ricin^CS^* | | 87.9 | ± 5.8 | 0.002 | | 86.3 | ± 6.1 | 10^-3^ |
| *hh-G4>GFP* | | 100 | ± 2.5 | - | | 100 | ± 2.4 | - |
| *hh-G4> Ricin^CS^* | | 85.7 | ± 2.5 | 10^-8^ | | 92 | ± 2.7 | 10^-3^ |
| *en-G4> Ricin^CS^ +* | *>p35* | 75 | ± 5 | 10^-7^ | 84 | | ± 5 | 10^-5^ |
|  | *Df(H99)* | 88 | ± 3 | 10^-5^ | 85 | | ± 2 | 10^-6^ |
|  | *>Diap1* | 80 | ± 3 | 10^-6^ | 84 | | ± 2 | 10^-7^ |
|  | *dronc^L29^/+* | 72 | ± 3 | 10^-8^ | 79 | | ± 5 | 10^-9^ |
|  | *>dp53^DN(CT))^* | 69 | ± 3 | 10^-4^ | 101 | | ± 6 | 0.922 |
|  | *>dp53^DN(259H)^* | 50 | ± 7 | 10^-13^ | 90 | | ± 1 | 10^-5^ |
|  | *>dp53^dsRNA^* | 68 | ± 7.9 | 10^-9^ | 101 | | ± 4.7 | 0.655 |
|  | *dp53^ns^* | lethal | - | - | - | | - | - |

###### Tissue size values of compartments expressing and not expressing the *Ricin^cs^*, *PTEN* or *4E-BP^AA^* transgenes and measured as a ratio (in percentage) with respect to control wings expressing GFP in the same domains (underlined). These values correspond to the average of 10 adult wings with their corresponding standard deviations. A t-test was carried out to calculate the p value as a measurement of the statistical significance of the difference between transgene expressing and GFP expressing wings.

**Table S2B**

| **Area of the transgene expressing and non-expressing territories (% of controls)** | | | | | | |
| --- | --- | --- | --- | --- | --- | --- |
| **Genotypes** | **Transgene expressing territory**  **(Area L2-L5)** | | **p-value** | **Transgene non-expressing territory (AWM-L2/L5-PWM)** | | **p-value** |
| *spalt^PE^-G4>GFP@30^o^C* | 100 | ± 3.1 | - | 100/100 | ± 6.9/± 6.3 | - |
| *spalt^PE^-G4>dMyc^dsRNA^@30^o^C* | 62 | ± 1.6 | 10^-7^ | 75/85 | ± 1.3/± 1.2 | 10^-5^/0.002 |

###### Tissue size values of wing territories expressing and not expressing a *dMyc^dsRNA^*, transgene and measured as a ratio (in percentage) with respect to control wings expressing GFP in the same domains (underlined). These values correspond to the average of 10 adult wings with their corresponding standard deviations. A t-test was carried out to calculate the p value as a measurement of the statistical significance of the difference between transgene expressing and GFP expressing wings. The transgene was expressed in the *spalt* domain (between longitudinal veins L2 and L5). The areas of the following neighboring domains were also measured: (1) AWM-L2: area between the anterior wing margin and longitudinal vein L2, and (2) L5-PWM: area between longitudinal vein L5 and the posterior wing margin).
