# Supplementary material for: A dp53-Dependent Mechanism Involved in Coordinating Tissue Growth in Drosophila
Source: PLoS Biol. 2010 Dec 14;8(12):e1000566. doi: 10.1371/journal.pbio.1000566 (PMC3001892; doi:10.1371/journal.pbio.1000566)
Supplement: Table S3 — Cell density values of compartments expressing and not expressing the Ricincs, PTEN, or 4E-BPAA transgenes and measured as a ratio (in percentage) with respect to control wings expressing GFP in the same domains (underlined). These values correspond to the average of 10 adult wings with their corresponding standard deviations. A t test was carried out to calculate the p value as a measurement of the statistical significance of the difference between transgene-expressing and GFP-expressing wings. (0.06 MB DOC) [file pbio.1000566.s008.doc]

**Table S3**

| **Cell Densities (% of controls)** | | | | | | | | |
| --- | --- | --- | --- | --- | --- | --- | --- | --- |
| **Genotypes** | | **Transgene expressing compartment** | | **p-value** | | **Transgene non-expressing compartment** | | **p-value** |
| *en-G4>GFP* | | 100 | ± 1.2 | - | | 100 | ± 2.0 | - |
| *en-G4> Ricin^CS^* | | 104 | ± 1 | 0.04 | | 103 | ± 2 | 0.04 |
| *en-G4>PTEN* | | - | - | - | | - | - | - |
| *en-G4>4E-BP^AA^* | | 108 | ± 2.7 | 10^-3^ | | 106 | ± 1.5 | 10^-4^ |
| *ci-G4>GFP* | | 100 | ± 2.1 | - | | 100 | ± 1.9 | - |
| *ci-G4> Ricin^CS^* | | 117.9 | ± 2.7 | 10^-11^ | | 111.6 | ± 2.2 | 10^-13^ |
| *ci-G4>PTEN* | | 119 | ± 3.1 | 10^-8^ | | 101 | ± 3.1 | 0.723 |
| *dpp-G4>GFP* | | 100 | ± 2.7 | - | | 100 | ± 2.6 | - |
| dpp-*G4> Ricin^CS^* | | 106.9 | ± 2.1 | 10^-3^ | | 107.4 | ± 1.3 | 10^-4^ |
| *ptc-G4>GFP* | | 100 | ± 2.9 | - | | 100 | ± 4.1 | - |
| *ptc-G4> Ricin^CS^* | | 97.1 | ± 3.5 | 0.123 | | 97.8 | ± 4.1 | 0.365 |
| *hh-G4>GFP* | | 100 | ± 3.0 | - | | 100 | ± 3.1 | - |
| *hh-G4> Ricin^CS^* | | 101.7 | ± 3.4 | 0.357 | | 103 | ± 2.7 | 10^-3^ |
| *en-G4> Ricin^CS^ +* | *>p35* | 121 | ± 1 | 10^-12^ | 115 | | ± 5 | 10^-5^ |
|  | *Df(H99)/+* | 104 | ± 2 | 0.06 | 120 | | ± 2 | 10^-9^ |
|  | *>Diap1* | 111 | ± 6 | 10^-6^ | 117 | | ± 1 | 10^-11^ |
|  | *dronc^L29^/+* | 110 | ± 5 | 0.04 | 115 | | ± 4 | 10^-5^ |
|  | *dp53^DN(CT)^* | 94 | ± 3 | 0.054 | 101 | | ± 2 | 0.725 |
|  | *dp53^DN(259H)^* | 105 | ± 1 | 10^-3^ | 93 | | ± 2 | 10^-4^ |
|  | *>dp53^dsRNA^* | 98 | ± 4 | 0.487 | 103 | | ± 4 | 0.036 |
|  | *dp53^ns^* | lethal | - | - | - | | - | - |

###### Cell density values of compartments expressing and not expressing the *Ricin^cs^*, *PTEN* or *4E-BP^AA^* transgenes and measured as a ratio (in percentage) with respect to control wings expressing GFP in the same domains (underlined). These values correspond to the average of 10 adult wings with their corresponding standard deviations. A t-test was carried out to calculate the p value as a measurement of the statistical significance of the difference between transgene expressing and GFP expressing wings.
