# Supplementary material for: A dp53-Dependent Mechanism Involved in Coordinating Tissue Growth in Drosophila
Source: PLoS Biol. 2010 Dec 14;8(12):e1000566. doi: 10.1371/journal.pbio.1000566 (PMC3001892; doi:10.1371/journal.pbio.1000566)
Supplement: Table S4 — Tissue size values of Ricincs -expressing and non-expressing compartments measured as a ratio (in percentage) with respect to control wings expressing GFP in the same domain (underlined). These values correspond to the average of 10 adult wings with their corresponding standard deviations. A t test was carried out to calculate the p value as a measurement of the statistical significance of the difference between transgene-expressing and GFP-expressing wings. Larvae were grown at 18°C and switched to 29°C at different times of development until adulthood. (0.04 MB DOC) [file pbio.1000566.s009.doc]

**Table S4**

| **Time-lapse experiment: Compartment Areas (% of controls)** | | | | | | | | | | |
| --- | --- | --- | --- | --- | --- | --- | --- | --- | --- | --- |
| **Genotypes** | **Induction at 29ºC** | **Transgene expressing compartment** | | **p-value** | **Transgene non-expressing compartment** | | **p-value** | **Total** | | **p-value** |
| *ci-G4>GFP* | *Early second instar* | 100 | ± 3.8 | - | 100 | ± 2.8 | - | 100 | ± 2 | - |
| *ci-G4> Ricin^CS^* | *Early second instar* | 63.2 | ± 4.7 | 10^-12^ | 67.5 | ± 6.3 | 10^-10^ | 66 | ± 4 | 10^-13^ |
|  | *Early third instar* | 78.1 | ± 4.9 | 10^-7^ | 80.4 | ± 5.2 | 10^-6^ | 80 | ± 5 | 10^-7^ |
|  | *Late third instar* | 81.6 | ± 5.6 | 10^-5^ | 86.1 | ± 3.7 | 10^-5^ | 86 | ± 4 | 10^-5^ |
|  | *Early Pupae* | 89.1 | ± 6.8 | 0.001 | 94.3 | ± 5.5 | 0.042 | 91 | ± 5 | 10^-3^ |

###### Tissue size values of *Ricin^cs^* expressing and non-expressing compartments measured as a ratio (in percentage) with respect to control wings expressing GFP in the same domain (underlined). These values correspond to the average of 10 adult wings with their corresponding standard deviations. A t-test was carried out to calculate the p value as a measurement of the statistical significance of the difference between transgene expressing and GFP expressing wings. Larvae were grown at 18*º*C and switched to 29*º*C at different times of development until adulthood.
