# Supplementary material for: A dp53-Dependent Mechanism Involved in Coordinating Tissue Growth in Drosophila
Source: PLoS Biol. 2010 Dec 14;8(12):e1000566. doi: 10.1371/journal.pbio.1000566 (PMC3001892; doi:10.1371/journal.pbio.1000566)
Supplement: Text S1 — Supporting Materials and Methods. (0.04 MB DOC) [file pbio.1000566.s010.doc]

**Text S1**

**Materials and Methods**

**Antibodies.**

Mouse anti-Wingless [1], mouse anti-Patched [2], rabbit anti-Spalt [3], guinea pig anti-Senseless [4]. Guinea-pig anti-dMyc [5].

***Drosophila* Strains.**

*UAS-dMyc^RNAi^* (ID 1419, VDRC), *UAS-hippo* (Flybase).

**Supporting References**

1. Brook WJ, Cohen SM (1996) Antagonistic Interactions Between Wingless and Decapentaplegic Responsible for Dorsal-Ventral Pattern in the Drosophila Leg. Science 273: 1373-1377.

2. Capdevila J, Estrada MP, Sánchez-Herrero E, Guerrero I (1994) The *Drosophila* segment polarity gene *patched* interacts with *decapentaplegic* in wing development. EMBO J 13: 71-82.

3. de Celis JF, Barrio R, Kafatos FC (1996) A gene complex acting downstream of *dpp* in  *Drosophila* wing morphogenesis. Nature 381: 421-424.

4. Nolo R, Abbott LA, Bellen HJ (2000) Senseless, a Zn finger transcription factor, is necessary and sufficient for sensory organ development in Drosophila. Cell 102: 349-362.

5. Herranz H, Perez L, Martin FA, Milan M (2008) A Wingless and Notch double-repression mechanism regulates G1-S transition in the Drosophila wing. Embo J 27: 1633-1645.
